# Comparative assessment of species identification methods for European *Salicornia* sources: a multifaceted approach employing morphology, nuclear DNA content, phylogenetic markers, RNA topology, and SSR fingerprinting

**DOI:** 10.3389/fpls.2025.1666009

**Published:** 2025-09-19

**Authors:** Andre Fussy, Samuele Austoni, Traud Winkelmann, Jutta Papenbrock

**Affiliations:** ^1^ Institute of Botany, Leibniz University Hannover, Hannover, Germany; ^2^ Department of Food, Environmental and Nutritional Sciences, University of Milan, Milano, Italy; ^3^ Institute of Plant Genetics, Section Reproduction and Development, Leibniz University Hannover, Hannover, Germany

**Keywords:** microsatellite marker fingerprinting, molecular markers, morphology, nuclear DNA content, *Salicornia*, species delimitation

## Abstract

Accurate identification of *Salicornia* species is a fundamental prerequisite for their potential usability and domestication. This study utilized a multifaceted methodological approach integrating morphological, cytogenetic, and molecular techniques to identify species from available European *Salicornia* sources. The following methods were compared: nuclear DNA content analysis; application of marker-based DNA barcoding via four common *Salicornia* markers; investigations of RNA topologies of these marker sequences by predicting theoretical secondary structures; utilization of diagnostic single-nucleotide polymorphism (SNP) positions within the external transcribed spacer (ETS) marker sequences for European *Salicornia* taxa; comparison of three promising microsatellite (SSR) markers regarding their ability to differentiate *Salicornia* subspecies; and evaluation of morphological data on habitus and flower characteristics utilizing a *Salicornia* identification key. The results demonstrate that ETS marker analysis offers reliable and cost-effective species determination, with SNP comparisons being more user friendly than phylogenetic trees are, and microsatellite markers can be differentiated down to the subspecies level via fragment length differences. However, microsatellite analysis alone is not suitable for primary species identification. DNA content can provide a rough estimation of potential species and is already more reliable than morphological methods. The differentiation among species is crucial for creating transparency for farmers and consumers and for initiating breeding processes, particularly within the context of frequent misidentification.

## 1 Introduction

Climate change is increasingly impacting agro-food systems, with factors such as extreme weather, drought, soil salinization, sea-level rise, and pests affecting agricultural land while the global population grows (Kumar et al., 2022). Despite past increases in food production, 828 million people faced hunger in 2022 (Wijerathna-Yapa and Pathirana, 2022). In general, the current annual yield increases of the top four crops are insufficient to double global production by 2050 to meet projected food demands, requiring spatially targeted efforts to increase crop yields (Ray et al., 2013). Soil salinity, sodicity, and edaphic conditions arising from natural and anthropogenic processes present key obstacles in this context. The progressive salinization and sodification of arable land pose significant threats to both environmental sustainability and agricultural productivity (Singh, 2015; Ivushkin et al., 2019). Agricultural practices, particularly irrigated cropping systems, demonstrably exacerbate the natural processes of soil salinization (Forkutsa et al., 2009; Litalien and Zeeb, 2020). The expansion of global drylands (Hassani et al., 2021) is establishing saline soils as a worldwide environmental challenge (Stavi et al., 2021). This is further exacerbated by the dramatic influence of changing climate patterns on vulnerable agricultural areas, including arid, semiarid and coastal zones (Corwin, 2021). Addressing the necessity of meeting increasing food demands under the growing challenges of climate change, *de novo* domestication - the direct domestication of resilient wild or underutilized plant species - presents a potentially efficacious and accelerated pathway toward future sustainable agricultural systems (Palmgren and Shabala, 2024).

Salt-tolerant plant species, so-called halophytes, have the potential to be used in saline agriculture to transform and restore key functions of salt-affected and marginal lands. *Salicornia*, a halophytic genus, offers significant potential for a closed-loop bioeconomy because of its salt tolerance, expanding cultivation to saline conditions and promoting efficient resource management (Ventura et al., 2015). In addition to its environmental benefits, it shows promise for use in medicine, as a biofuel, and as a nutritious source for humans and animals (Cárdenas-Pérez et al., 2021). Therefore, *Salicornia* species could be new salt-tolerant crop plants (Bazihizina et al., 2024). Modern water-based systems, such as hydroponics, which are powered by renewable energy, enable sustainable, climatically independent regional crop production and enhance food security (Fussy and Papenbrock, 2022). Hydroponic cultivation also facilitates optimal salinity control, for example, for the use of brackish water, with concentrations of approximately 15 g/L or 250 mM NaCl reported for optimal *Salicornia europaea* biomass production (Turcios et al., 2023; Fussy and Papenbrock, 2024). Despite the promising cultivation of members of the genus *Salicornia* as new horticultural crop plants, conclusive taxonomic studies of *Salicornia* are limited by its unique morphology, broad phenotypic plasticity, and few distinguishing characteristics (Kadereit et al., 2007). Accurate classification thus requires molecular analysis relying on reference sequence data. Well-curated public databases, ensuring consistent classification where sequences of the same species are congruent and distinct from those of other species, are essential for reliable phylogenetic analyses and the avoidance of misleading inferences (Liu et al., 2011; Cerutti-Pereyra et al., 2012).

The commercial cultivation of *Salicornia* in Europe faces challenges due to unclear species identities. This lack of clarity can lead to inconsistent crop performance, difficulties in optimizing cultivation practices for specific species, and mislabeling in the market, ultimately affecting product quality and consumer trust. Various species are marketed as *S. europaea*”, leading to ambiguity in seed sourcing and labeling, hindering farmers and breeders from requiring accurate species information for cultivation, hybridization or selection. Varying reproductive isolation and species-specific traits necessitate this knowledge for targeted breeding (Bazihizina et al., 2024).

Advances in biotechnological tools, including molecular markers and gene banks, support the precise characterization and conservation of genetic resources, thereby contributing to the maintenance of diversity and their sustainable utilization for global food security (Salgotra and Chauhan, 2023). Coordinated DNA-based species identification methods, such as nrDNA and cpDNA barcoding, as demonstrated by improved taxonomic understanding in *Salicornia* spp., are both recognized as necessary (Cristescu, 2014) and already yielding results (Kadereit et al., 2012; Jamdade et al., 2022; Janssen et al., 2023; Sciuto et al., 2023). The current ambiguous nomenclature necessitates a robust, standardized identification method, potentially relying on molecular techniques for accurate differentiation at the species and subspecies levels, with breeding programs possibly requiring finer resolution than commercial production (Siridewa et al., 2024). While traditional morphological identification and targeted barcoding approaches are effective for species discrimination, advanced molecular approaches such as genome-wide association studies offer powerful avenues for identifying genetic loci underlying qualitative traits (Visscher et al., 2017; He and Gai, 2023), including those relevant for species identification or ecological adaptations within complex genera such as *Salicornia*.

With respect to the current state of knowledge, a number of inconsistencies in the naming of species and in the sequence labeling of database entries have already been revised (Kadereit et al., 2012; Piirainen et al., 2017), which has led to a significant improvement in the taxonomic presentation of the species. Specifically, a revised taxonomy for the European *Salicornia* species, within which two distinct groups are identified, represents the current state of knowledge (Kadereit et al., 2012). Chromosome numbers and genome sizes, which are largely determined by flow cytometry, are used to differentiate species, particularly within taxonomically challenging genera (Albach and Daubert, 2024). For *Salicornia*, the mainly diploid group (2n = 18) included two species (*S. perennans* Willd. and *S. europaea*) and five subspecies (*europaea, disarticulata, marshallii, perennans, altaica*) (Kadereit et al., 2012). Conversely, the mainly tetraploid group (2n = 36) comprises two species (*S. persica* Akhani and *S. procumbens* Sm.) and six subspecies (*procumbens* (syn. *dolichostachya*), *freitagii, heterantha, pojarkovae, persica* and *iranica*) (Kadereit et al., 2012). Notably, both groups may include species with mixed ploidy levels, such as the diploids *S. heterantha* and *S. iranica* within the *S. procumbens* clade and the decaploid (2n = 90) *S. altaica* in the *S. europaea* clade (Akhani, 2003; Beer and Demina, 2005; Ломоносова, 2005). In addition, a number of investigations have quantified the nuclear DNA content across different Salicornioideae species, revealing further variations in their haploid genomic complement (Shepherd and Yan, 2003; Koce et al., 2008; La Fuente et al., 2016; Zonneveld, 2019). Ploidy levels combined with nuclear DNA contents thus offer initial insights into potential species classifications.

The broad barcode framework for Salicornioidae was developed as a result of extensive phylogenetic investigations that sought to address various research questions (Kadereit et al., 2012; Singh et al., 2014; Steffen et al., 2015; Piirainen et al., 2017; Jamdade et al., 2022; Sciuto et al., 2023). The requirements for the variability of the barcoding regions differ depending on the objective of the studies. While the external transcribed spacer (ETS) rDNA region alone may not be suitable for discriminating between subspecies, it has proven to be a valuable tool for phylogenetic analyses and sequence-based species comparisons within *Salicornia* and related genera in the Salicornioideae subfamily (Kadereit et al., 2012; Singh et al., 2014; Sciuto et al., 2023). Furthermore, the use of a combination of ETS and internal transcribed spacer (ITS) rDNA regions was found to provide the highest discriminatory power between populations of one *Salicornia* species, followed by the use of the *maturase K* (*matK*)-cpDNA region (Jamdade et al., 2022). The *matK* gene, located in the chloroplast genome, has a high mutation rate and high discriminatory power, allowing it to be used as a DNA barcode region in phylogenetic analyses of plants (Hollingsworth et al., 2009). Similarly, the spacer between the ATP synthase beta subunit and the large subunit of ribulose-1,5-bisphosphate carboxylase/oxygenase genes (*atpB-rbcL*), a noncoding region within chloroplast DNA, has served as another commonly used molecular marker in various phylogenetic analyses of Salicornioideae (Steffen et al., 2015; Piirainen et al., 2017; Jamdade et al., 2022). On the basis of numerous previous barcode-based studies of *Salicornia* and other relatives within the Chenopodiaceae family, there is an extensive pool of sequences of diverse nrDNA, cpDNA and mitochondrial DNA (mtDNA) regions, with the largest pool of compatible sequences existing for the ETS region within nrDNA (NCBI, 2024). The most frequently used and promising barcodes are compared in the current investigation to highlight uncertainties as well as their applicability.

Phylogenetic tree creation relies on the principle of sequence homology, where sequence polymorphisms indicate evolutionary divergence. Consequently, variations in alignments of representative DNA sequences can be used to distinguish species, bypassing the need for computationally intensive phylogenetic algorithms. Such barcoding sequence alignments can provide diagnostic single-nucleotide polymorphism (ETS) positions with sufficient variability and conservation for clear delimitation of genera, species, and partly subspecies (Kadereit et al., 2012; Schroeder, 2018). Compared with whole-sequence comparisons, RNA topology analysis offers a more straightforward evaluation of polymorphisms (including SNPs), leveraging the relationship between sequences and the three-dimensional structure of a DNA-to-RNA translated sequence (Keller et al., 2008; Schultz and Wolf, 2009). This strategy offers a valuable alternative to the analysis of individual SNPs within extensive sequence alignments. The key advantages of RNA topology analysis are its comprehensive sequence illustration compared with sequence data alone and the conserved nature of RNA secondary structures within a species, making it a reliable tool for species delimitation (Keller et al., 2010; Yang et al., 2018). This can be viewed as a beneficial reduction in data complexity. Furthermore, microsatellite markers (SSRs) have proven valuable in molecular taxonomy, offering high polymorphism, codominant inheritance, and reproducibility at relatively low cost (Vanderpoorten et al., 2011). In *Salicornia*, SSR markers, on the basis of their length differences, may reveal subtle differences between species and subspecies (Hamm et al., 2023; Liang et al., 2025).

This study assesses the application-oriented effectiveness of different genetic determination approaches for *Salicornia* species compared with traditional methods of identification on the basis of morphological criteria. For this purpose, *Salicornia* seeds from 11 European suppliers were acquired, representing a broad range of sources at the time of investigation. The primary objective is to identify methods that enable easy, accurate, and cost-effective classification of unknown *Salicornia* samples, thereby providing practical tools for industry and research.

Therefore, whether the construction of phylogenetic trees on the basis of single-barcode markers represents a methodology sufficient for species identification on the basis of reference sequences should be evaluated. Furthermore, the level of detail of this approach might be increased by comparing diagnostic single nucleotide polymorphisms (SNPs) and RNA topology variations. In addition, nuclear DNA content determination might provide a first indication of species groups by linking it to known genome sizes and ploidy levels. In contrast, the benefit of SSR fingerprinting might be highly marker-dependent and might provide the highest level of detail for subspecies differentiation. Ultimately, various identification options tailored to different levels of detail and prior knowledge are recommended for suppliers and breeders to select from, with a focus on classification methods of unknown *Salicornia* sources that are both easy and accurate as well as affordable.

## 2 Materials and methods

### 2.1 Plant material

Seeds were obtained from various suppliers, with different seed stocks indicated by numbers for anonymity. All information regarding the original indication of the species and the country of origin of the ordered seed samples were documented (**Table 1**). The seeds were sown and grown in a mixture of sand and soil (Einheitserde Classic Profi, Balster Einheitserdewerk GmbH, Fröndenberg, Germany) in a greenhouse under controlled conditions as previously described (Turcios et al., 2023). Given the highly variable germination rates among seed samples, approximately 200 seeds per seed stock were sown (June 10, 2024), and once seedlings of at least 5 cm height were established in all growth containers, plants of similar size per seed stock were selected for DNA extraction (with five plants per supplier labeled with a-e). Two additional plants per supplier were transferred to separate pots to monitor development and morphology during the vegetative phase. A further three plants per supplier were initially isolated in pots and transferred to short-day conditions (9 h photoperiod) for 3 months to induce flowering. The remaining plants were left to grow for 3–6 months to obtain fresh shoot tips for flow cytometry.

**Table 1:** Summary of samples obtained from different suppliers and cultivated for this study.

Supplier	Indicated species	Country of sale
1	*S. europaea* sample 1	Belgium
2	*S. ramosissima* sample 1	Portugal
3	*S. bigelovii*	Belgium
4	*S. ramosissima* sample 2	France
5	*S. fruticosa*	Portugal
6	*S. europaea* sample 2	Netherlands
7	*S. europaea*/*Sarcocornia fruticosa*	Portugal
8	*S. europaea* sample 3	Belgium
9	*S. europaea* sample 4	Ireland
10	*S. europaea* sample 5	France
11	*S. ramosissima* sample 3	Portugal

Supplier: number given to a specific seed stock; Indicated species: originally declared species name and ascending sample numbering for multiple identical names; Location: country of origin of the seeds.

### 2.2 Morphological studies of the habitus and flower structure

To identify potential species-specific morphological characteristics, a detailed examination of the habitus and floral structures of mature plants was conducted for each supplier, following previously published methods (Kadereit et al., 2012). As the published dichotomous key (Piirainen et al., 2017) relies on floral traits and flower induction was not possible for suppliers 5 and 7, morphological comparisons of these accessions were limited to photographic documentation of magnified shoots and branching points. These images are presented alongside floral images obtained for the other flowering samples, with all floral photographs captured at a comparable developmental stage. Habitus assessments, however, encompassed plants of all eleven suppliers, with two individual plants per stock photographed at three distinct developmental stages: early (August 27, 2024), middle (October 10, 2024), and late (December 6, 2024). This analysis facilitated the observation of ontogenetic changes in plant architecture and overall morphology across the different suppliers.

### 2.3 DNA isolation, amplification, and sequencing

A previously described method (Singh et al., 2014) was used for DNA extraction with modifications: Young stems of plants from all suppliers (**Table 1**) were homogenized in liquid nitrogen using a mortar and pestle. Subsequently, genomic DNA was extracted from 100 mg of the resulting fine powder via the Plant Nucleospin II Kit (Macherey & Nagel, Düren, Germany) following the manufacturer’s instructions for plants via the CTAB lysis buffer system. The concentration of the extracted DNA was determined photometrically via a SynergyMx plate reader (BioTek Instruments, Winooski, USA) with a TAKE-3 microvolume plate (BioTek Instruments). Its quality was assessed via gel electrophoresis via 1.5% agarose stained with MIDORI Green Advance (NIPPON Genetics EUROPE GmbH, Düren, Germany). The ratio of the absorbance at 260 nm to that at 230 nm (ideally between 2.0 and 2.2) and the ratio at 260 nm to that at 280 nm (ideally ≥ 1.7) were also controlled (Heptinstall and Rapley, 2000). Only DNA isolates demonstrating these required purity ratios and exhibiting nonfragmented bands on agarose gel electrophoresis were selected for subsequent analyses.

All PCR amplifications for phylogenetic barcoding markers were performed in a lid-heated thermocycler (peqSTAR XS, VWR International GmbH, Darmstadt, Germany) with 25 µl reaction volumes, including 0.2 mM dNTPs (dNTP-Set, Life Technologies GmbH, Darmstadt, Germany), 1 U *Taq* polymerase and 10× standard *Taq* reaction buffer (both New England Biolabs, Ipswich, MA, USA), 30–100 ng template DNA and 0.2 µM of each primer (Eurofins Genomics GmbH, Ebersberg, Germany). The nuclear ETS region was amplified via the primer pair “18S-II-rev” (Ochsmann, 1998) and the slightly modified “ETS-Salicornia” (Kadereit et al., 2007). The nuclear ITS region was amplified via the primer pair “ITS-28S” and “ITS-18S” (Kadereit et al., 2006). The nuclear *matK–trnK* region of the cpDNA was amplified via the primer pair “MatK–1RKIM–f” and “MatK–3FKIM-r” (Kuzmina et al., 2012). The *atpB-rbcL* region of the cpDNA was amplified via the primer pair “*atpB*-*rbcL*-F” and “*atpB*-*rbcL*-R” (Xu et al., 2000). The primer sequences and individual PCR conditions are summarized in an additional file (**Supplementary Table S1**).

To evaluate the length, specificity, and quantity of each PCR product, all reactions were separated on 1.5% agarose gels stained with MIDORI Green Advance. Direct sequencing of the PCR product was performed by Microsynth (Microsynth AG, Göttingen, Germany).

### 2.4 Sequence alignment and tree construction

All subsequent analyses of the sequence data were conducted within the CLC Main Workbench (version 23.0.4, Qiagen, Venlo, Netherlands) environment, except where noted. Consensus sequences for both our generated and NCBI reference sequences were obtained, if needed, through reverse complementation. For sequences generated by Microsynth, initial quality assessment of the.ab1 files was performed, and all ambiguous terminal regions were trimmed. Following alignment with subsets of reference sequences [gap cost settings: gap opening cost = 10; gap extension cost = 1; free end gap cost], a minimal consensus sequence length was determined on the basis of a trade-off between the number of sufficiently long reference sequences and the maximum overall length of each sequence. For *Salicornia* spp. ETS marker references, only the previously annotated reference sequences from Kadereit et al. (2012) were used. The reference dataset was expanded for determining suppliers 5 and 7, including ETS sequences from *Sarcocornia* sp. and *Arthrocnemum* sp., as well as closely related species, namely, *Microcnemum coralloides* subsp. *coralloides*, *Tecticornia australasica*, and *Halosarcia indica* subsp. *bidens* reference sequences. To simplify the phylogenetic tree, redundant sequences of the same ribotype were subsequently hidden. To identify redundant, too short and inaccurate sequences of all available data for ETS, ITS, *matK* and *atpB-rbcL*, reference data were subjected to sequence alignments and comparisons. Sequences were considered redundant if they were 100% identical to another reference sequence, including the species name. However, redundant sequences from NCBI with different species names were retained, taking a lack of genetic variation, synonyms or imprecisely determined plant samples into account. All reference sequences for ETS, ITS, *matK*, and *atpB*-*rcL* used in the final trees and partial RNA structure predictions, including GenBank-IDs, vouchers, and species names, are documented in an additional file (**Supplementary Table S2**).

The alignments of our generated sequences and the processed reference datasets were analyzed for the best nucleotide substitution model in the maximum likelihood reconstruction. The analyses involved the application of the hLRT, BIC, AIC, and AICc model testing algorithms. The best fitting algorithm for phylogenetic reconstruction was determined on the basis of the results of these analyses. Phylogenetic analyses were conducted via maximum likelihood with UPGMA tree construction. The general time reversible (GTR) model with rate and topology variation was employed for ETS and ITS, whereas the Felsenstein 81 model with rate variation was used for *matK* and *atpB*-*rbcL*. To control the reliability of the phylogenetic trees, a bootstrap analysis with 1,000 replicates was conducted. Clades containing the species of interest were identified, assuming that samples within the same region or connected by the same branch likely belong to the same species. To simplify the main figures, we reduced the number of reference sequences by eliminating irrelevant species or species only distantly related to our target species while preserving clades of key species as well as contradictory connections of reference sequences for comparison and quality assessment. However, for the initial identification of the species, a broader perspective, considering all available and reliable reference sequences for a specific barcode, was taken.

### 2.5 SNP analysis

SNP analysis was applied exclusively to the ETS sequences because of the absence of clear references for the other markers. A previously published SNP comparison of the ETS region (Kadereit et al., 2012) provided an alignment of representative core sequences of *S. europaea*, *S. perennans*, *S. procumbens*, and *S. persica*, along with the outgroup *Salicornia* sp. (*S.* “crassa” group), including variable and diagnostic positions. Following this method, the generated ETS sequences were analyzed for homologies at these verified species-specific locations. Representative sequences were selected from the consensus of the five sequences obtained from each supplier. However, owing to substantial heterogeneity among the five samples of supplier 3, all individual sequences from this supplier were presented.

### 2.6 RNA topology analysis

To elucidate the phylogenetic relationships among *Salicornia* species, an in-depth analysis of RNA topology was performed following the methods of previous studies (Keller et al., 2008; Schultz and Wolf, 2009). The underlying hypothesis is that SNPs can induce variations in the predicted RNA secondary structure, thereby creating distinct topological profiles for each species. For this purpose, the sequences and reference sequence sets for the markers ETS, ITS, *matK*, and *atpB-rbcL*, which were already generated in 2.3 - 2.4, were used.

The primary objective of this computational approach using the CLC Main Workbench was to enhance the visualization and interpretation of SNPs, including gaps and ambiguous base pairs. For each supplier, RNA secondary structures of reference sequences from various species exhibiting congruence were grouped in vertically aligned clusters alongside the corresponding supplier sequences. This arrangement facilitated the assessment of species discrimination and the potential of the associated marker. To ensure clarity in individual analyses, a single, representative RNA secondary structure was chosen for each supplier. This selection was based on the fact that it was overwhelmingly supported by other predictions for the same supplier. Where different suppliers yielded identical structures, these structures were consolidated into a unified representation.

To facilitate comprehension of the RNA secondary structure, a color-coded system was implemented. Specific RNA motifs are represented as follows:

- Stems (black): These structures denote base-paired helical regions, forming the fundamental building blocks of the RNA tertiary structure.
- Hairpin loops (blue): These motifs consist of internal loops enclosed by two stem regions.
- Bulges (green): Unpaired nucleotides positioned within a helical stem are indicated in green.
- Interior loops (yellow): Internal loops not flanked by complete stems on both sides are depicted in yellow.
- Multiloops (red): Loops with more than two branches are represented in red.

### 2.7 Nuclear DNA content and ploidy estimation

The youngest stem segments (or leaves for reference plants) were sampled from mature specimens. The total nuclear DNA content of the stem tissue was determined via flow cytometry via either propidium iodide (PI) or 4’,6-diamidino-2-phenylindole (DAPI) staining, following established protocols (Bohanec, 2003; Dolezel et al., 2007). Propidium iodide staining was performed with *Lycopersicon esculentum* Mill. convar. infiniens Lehm. var. flammatum Lehm. ‘Stupicke Rane’ (Gene Bank Gatersleben acc. No. LYC 418) [1.96 pg/2C] as the internal standard, was exclusively employed for precise DNA content estimation of *Trifolium pratense* ‘Bingenheimer’. For *Salicornia* species, *T. pratense* served as the internal standard for DAPI staining, while *Glycine max* (L.) Merr. convar. max var. max ‘Cina 5202’ (Gene Bank Gatersleben acc. No. SOJA 392) [2.23 pg/2C] was used for *Sarcocornia fruticosa* and *Arthrocnemum macrostachyum*. Nuclei were initially extracted separately for reference peak profiling and subsequently coextracted by chopping with a razor blade in the manufacturer’s recommended nuclei extraction buffer (CyStain^®^ PI Absolute P or CyStain UV Precise P Automate, Sysmex Deutschland GmbH, Norderstedt, Germany). The resulting suspension was filtered through a 30 μm nylon mesh, and 2 ml of the respective staining solution was added. Flow cytometric measurements were performed on a Sysmex XF-1600™ flow cytometer (Sysmex Deutschland GmbH, Norderstedt, Germany) (linear scale), which acquired data for ten thousand nuclei from the primary peak per sample. The G0/G1 peak positions of the standard and target accessions were calculated via CyView and CyBatch software (Sysmex Deutschland GmbH).

### 2.8 Fragment length analysis of nuclear microsatellite loci via SSR markers

The extracted DNA of the five samples from the 11 suppliers was amplified via three previously reported promising nuclear microsatellite loci markers (Vanderpoorten et al., 2009). All PCR amplifications were performed in a lid-heated thermocycler (peqSTAR XS) with 100 µl reaction volumes including 0.2 mM dNTPs (dNTP-Set, Life Technologies GmbH), 1 U *Taq* polymerase and 10× Standard *Taq* reaction buffer (New England Biolabs), 100–400 ng template DNA and 0.4 µM of each primer (Eurofins Genomics GmbH). The microsatellite regions were amplified with primers for the previously published S2, S5, and S19 loci (Vanderpoorten et al., 2009), where each forward marker was labeled with a fluorophore suitable for multiplexing (**Supplementary Table S1**). Amplification was carried out under slightly modified conditions (**Supplementary Table S1**). Gel electrophoresis of the amplicons revealed no specific fragments for samples from suppliers 5 and 7, which were subsequently excluded from further processing. The PCR products were purified via the NucleoSpin™ Gel and PCR Clean-up Kit (Macherey & Nagel), which employs 50% diluted NTI buffer to remove small fragments such as primers and excess dNTPs. The DNA concentration of the purified PCR products was subsequently photometrically determined to combine all three markers at the same concentration in 50 µl volumes per sample, according to Eurofins’ specifications. Five samples from each of the suppliers where clear amplicons could be generated were subsequently forwarded to Eurofins for analysis for fragment length analysis, and the internal lane standard (ILS-600) was added.

## 3 Results

### 3.1 Morphological observations

**Figure 1:**
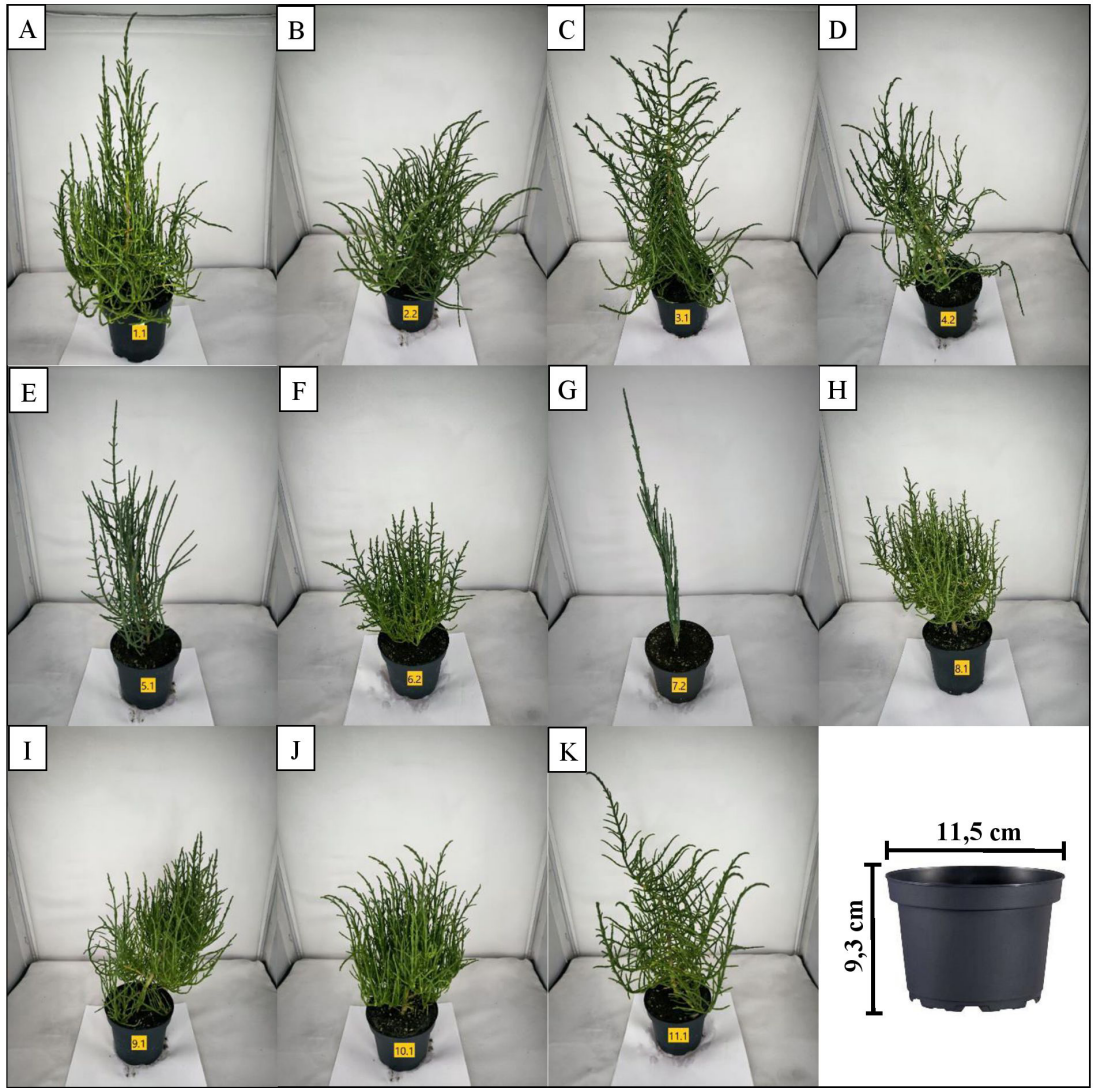
Mid-growth habitus of representative plants per supplier: **(A)** Supplier 1, **(B)** Supplier 2, **(C)** Supplier 3, **(D)** Supplier 4, **(E)** Supplier 5, **(F)** Supplier 6, **(G)** Supplier 7, **(H)** Supplier 8, **(I)** Supplier 9, **(J)** Supplier 10, and **(K)** Supplier 11.

**Figure 2:**
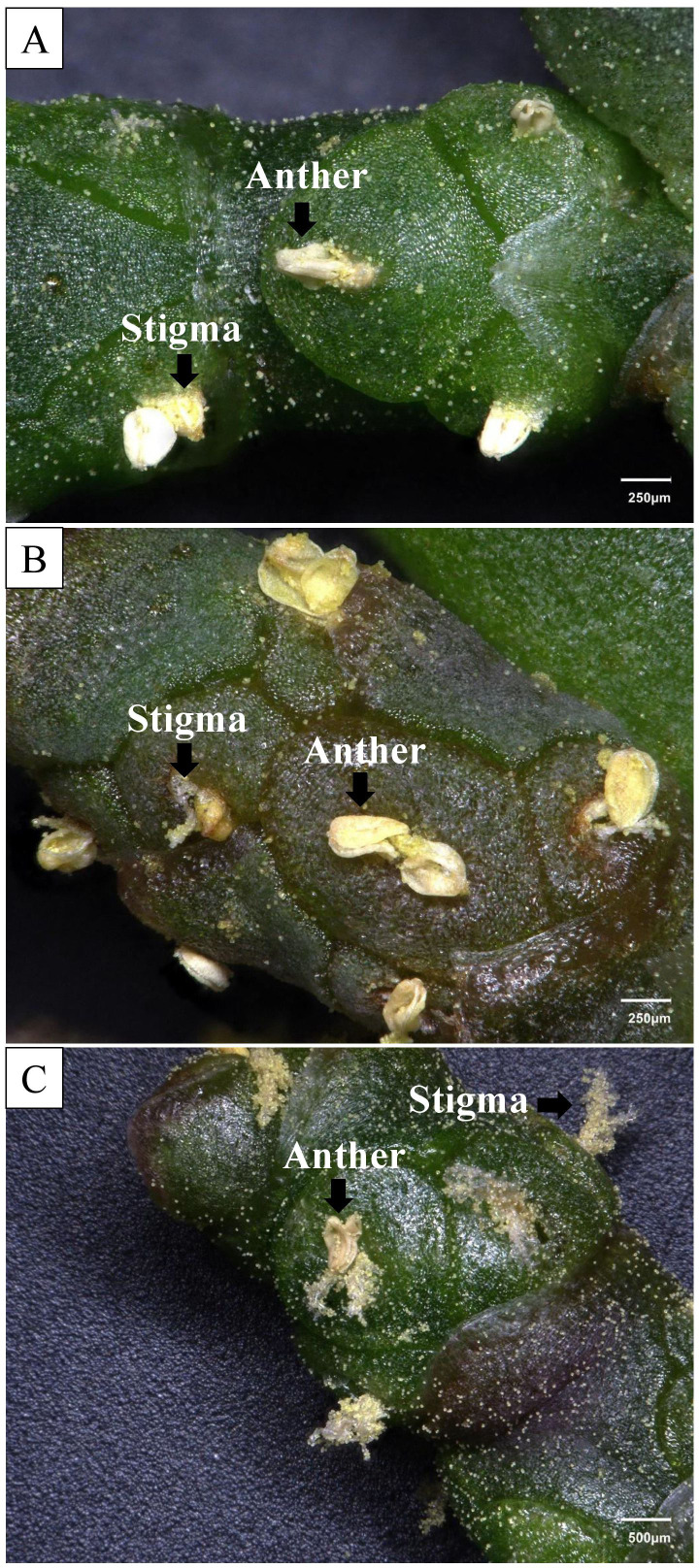
Flower details of the three main taxa: **(A)** Supplier 2, 100x; **(B)** Supplier 9, 100x; and **(C)** Supplier 4, 50x. All the plants presented three flowers per cyme with simultaneously emerging anthers and stigmas.

Morphological examination of the habitus (**Figure 1**) and flowers (**Figure 2**) from suppliers 1, 2, 3, 6, 8, 9, and 10 revealed only a few discernible differences. All habitus images, depicting two plants per supplier throughout the three growth stages, are summarized in an additional file (**Supplementary Table S3**). Another additional file compiles all images showing flower details at various magnifications of the plants from all suppliers, contingent on successful flowering induction (**Supplementary Table S4**). On the basis of vegetative and flower characteristics, tentative species assignments of suppliers 1, 2, 3, 6, 8, 9, and 10 to *Salicornia europaea* subsp. *europaea* or *Salicornia perennans* subsp. *perennans* were made utilizing a previously published identification key (Kadereit et al., 2012). Classification on the basis of the species identification key was limited to two primary characteristics: “Flowers consistently 3 per cyme, with the visible portion of lateral flowers smaller than that of the central flower” (**Figure 2**) and “fertile spikes usually shorter than the rest of the plant.” These criteria are applicable to *S. persica*, *S. europaea*, and *S. perennans*.

Independent of the identification key application, differences in growth habits were noted among these groups. Suppliers 1 and 8 exhibited denser growth than did suppliers 2, 3, and 11 (**Figure 1**). Furthermore, all the plants of these suppliers (1, 2, 3, 8, 11) presented a more upright growth form with a greater overall height than did the plants of suppliers 6, 9, and 10, the latter of which presented predominantly narrow and bushy development (**Figure 1**). A somewhat strict arborescent or pyramidal growth form with a clear main stem and distinctly decussated lateral branches further characterized the habitus of the plants from supplier 3 (**Figure 1**).

Plants from supplier 4 were characterized by an inflorescence axis that was consistently and significantly longer than those observed in the previously described suppliers, often accompanied by predominantly irregular growth forms (**Figure 1**). These traits align with the characteristics of *Salicornia procumbens* subsp. *procumbens* (Kadereit et al., 2012). Confirming features included the perianth tube of all flowers being sunken but clearly separate from the fleshy inflorescence axis, and flowers arranged “3 per cyme, visible part of lateral and central flowers subequal in size”. Furthermore, another morphological feature of supplier 4 was the comparatively larger size of its flowers (**Figure 2**).

Morphological classification of suppliers 5 and 7 via a previously published identification key (Piirainen et al., 2017) was not feasible because of the absence of flowering. Alternatively, growth habits were evaluated to distinguish these samples. Both supplier 5 and supplier 7 exhibited a strong upright growth habit with a distinct main stem and strictly ascending, long lateral branches, which contrasts with the narrow-growth types (suppliers 6, 9, and 10) and shows a more strictly ascending habit than the more numerous and widely spreading lateral branches observed in supplier 4 (**Figure 1**). Furthermore, variations in stem coloration were observed. Supplier 5 presented a darker, more intense green hue, whereas supplier 7 presented a glaucous (blue–green) appearance (**Figure 1**). The remaining suppliers generally displayed lighter green tones. Overall, while distinctions in growth form were observed between suppliers 1, 2, 3, 8, and 11 compared with suppliers 6, 9, and 10 and further from suppliers 5 and 7, a definitive taxonomic assignment for any of these groups using identification keys was not possible. Only the distinct characteristics of Supplier 4 strongly suggested an assignment to *S. procumbens* subsp. *procumbens*.

### 3.2 Tree evaluation

#### 3.2.1 ETS tree evaluation

**Figure 3:**
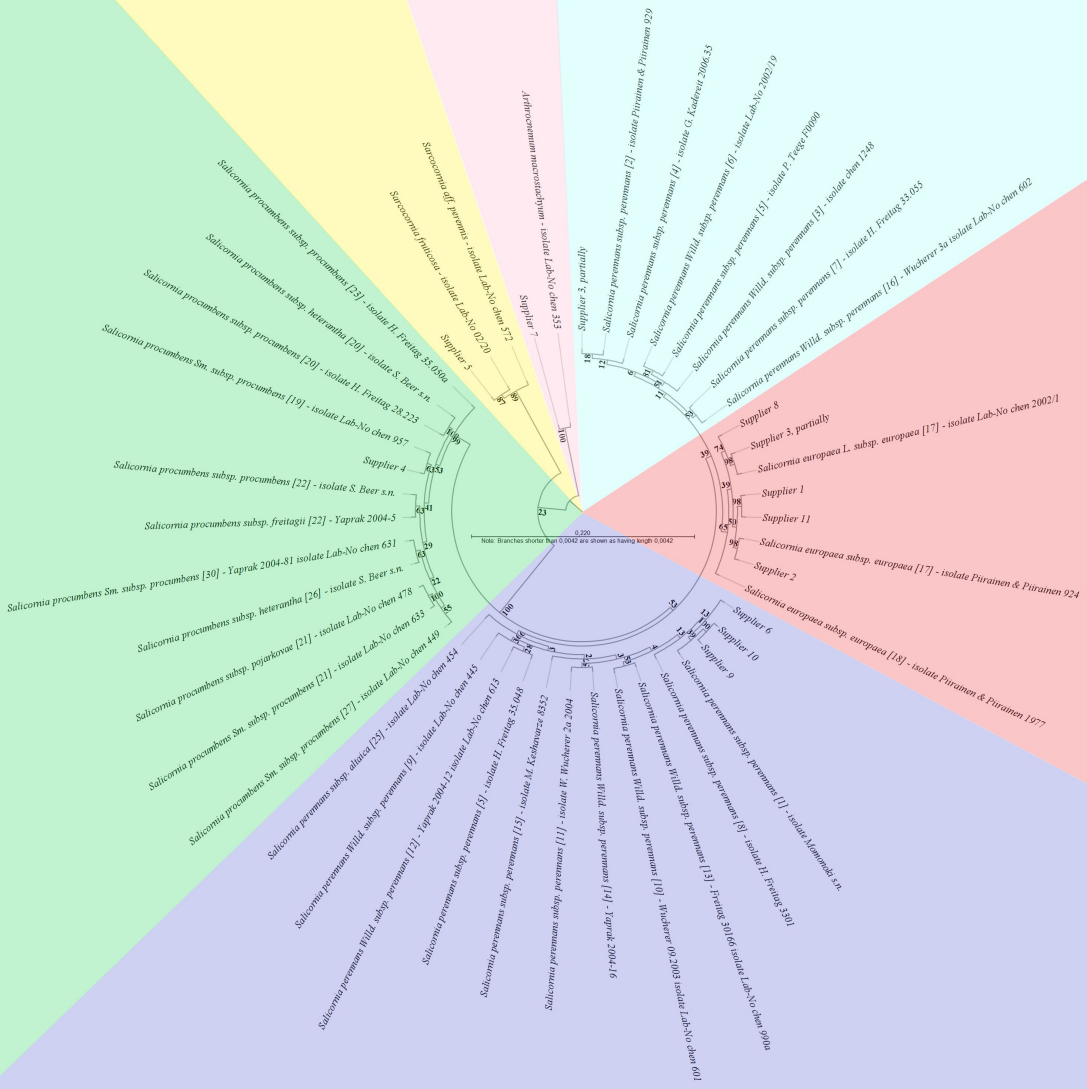
The rooted maximum-likelihood (ML) phylogenetic tree was inferred from the alignment of the genomic external transcribed spacer and small subunit ribosomal RNA partial sequences under the best-fit model GTR+G+T (selected by CLC Main Workbench hLRT, BIC, AIC, and AICc model testing). Sequence inclusion was limited to essential *Salicornia* ribotypes and reference sequences from *Sarcocornia* and *Arthrocnemum*, which facilitated the identification of supplier sequences. The bootstrap values from 1000 resamplings are given at each node, and the branch lengths are stretched for easier representation, as indicated in the scale.

To evaluate the phylogenetic trees, attention was focused on the congruent occurrence of individual species (including synonymous species names) within clades, including bootstrap probabilities. The complete phylogenetic tree of the ETS sequences, including all non-redundant sequences, is presented as an additional file (**Supplementary Table S7**; **Figure 1**). Analyzing the ETS phylogenetic output (**Figure 3**), the sequences from suppliers 1, 2, 8, and 11, along with some samples from supplier 3, clustered distinctly and with moderate support (65% bootstrap probability) exclusively with reference sequences of *Salicornia europaea* L. subsp. *europaea* (ribotype 17), indicating their assignment to this subspecies. Interestingly, as **Figure 3** shows, two other samples from supplier 3 aligned with a separate clade composed solely of *Salicornia perennans* subsp. *perennans* reference sequences (ribotypes 2, 3, 4, 5, 6, 7, and 16). However, the support for this clades’s distinctness was insufficient (11% bootstrap probability). From **Figure 3**, it is evident that the sequences from supplier 4 were found within a highly supported (100% bootstrap probability) clade containing only *S. procumbens* species, and within this cluster, the samples from supplier 4 were most closely related to those from *Salicornia procumbens* subsp. *procumbens* (ribotype 19), albeit with only low support (41% bootstrap probability). For supplier 5, another clade (89% bootstrap probability) was observed in **Figure 3**. Furthermore, its phylogenetic distance from other *Sarcocornia* species suggests a relationship with either *Sarcocornia fruticosa* or *Sarcocornia aff. perennis* (**Supplementary Table S7**; **Figure 1**). **Figure 3** also illustrates that the sequences from suppliers 6, 9, and 10 resided in a second clade (66% bootstrap probability) of *Salicornia perennans* subspecies reference sequences (including ribotypes 1, 5, 8, 9, 10, 11, 12, 13, 14, 15, and 25), showing the closest affinity to *Salicornia perennans* subsp. *perennans* (ribotype 1). Finally, sequences from supplier 7 formed a well-supported (81% bootstrap probability) clade with *Arthrocnemum macrostachyum*, distinct from other genera (**Supplementary Table S7**; **Figure 1**). Overall, suppliers 1, 2, 8, and 11 were largely assigned to *Salicornia europaea* subsp. *europaea*, whereas supplier 4 was unambiguously identified as *S. procumbens* subsp. *procumbens*. Suppliers 6, 9, and 10 were attributed to *S. perennans* subsp. *perennans* with reasonable certainty. Supplier 5 was definitively categorized as a *Sarcocornia* species (either *S. fruticosa* or *S. perennis*), and supplier 7 was categorized as *Arthrocnemum macrostachyum*. Notably, supplier 3 could not be definitively assigned to either *S. europaea* subsp. *europaea* or *S. perennans* subsp. *perennans*.

#### 3.2.2 ITS tree evaluation

The phylogenetic tree derived from the ITS marker is available in an additional file (**Supplementary Table S7**; **Figure 2**). The analysis placed sequences from suppliers 1, 2, and 11, along with some sequences from suppliers 3 and 8, within a clade supported by a 50% bootstrap probability. This clade included reference sequences of *S. disarticulata*, *S. x marshallii*, *S. obscura*, and *S. ramosissima*. The remaining sequences from supplier 8 were located in the nearest cluster, which is not clearly distinct from the first clade and contains *S. obscura*, *S. ramosissima*, and *S. x marshallii* reference sequences. These two clusters together show only weak differentiation (35% bootstrap probability) from a neighboring clade that includes the remaining two sequences from supplier 3, as well as the reference sequences of *S. ramosissima*, *S. europaea*, *S. prostata*, *S. patula*, *S. herbacea*, *S. persica*, and *S. perennans*. Notably, these three clusters collectively form a highly supported distinct group (98%) compared with the nearest cluster, which contains the sequences of suppliers 6, 9, and 10 without any further reference sequences. In contrast, all sequences from supplier 4 were within a clearly distinct clade (100% bootstrap probability) that also included reference sequences of *S. emerici*, *S. fragilis*, *S. procumbens*, and *S. dolichostachya*. For supplier 5, stable assignment to a clade composed solely of *Sarcocornia* species, specifically with sequences of *S. fruticosa*, *S. pruinosa*, *S. alpini*, *S. perennis*, and *S. lagascae*, was observed. Finally, the sequences from supplier 7, which were the most distant from the *Salicornia* clusters, were clearly assigned to *Arthrocnemum macrostachyum* within a well-separated clade (100% bootstrap probability). In summary, the ITS marker is insufficient for subspecies identification because the necessary reference data is unavailable. Suppliers 1, 2, 8, and 11 were assigned to *S. europaea*. While suppliers 6, 9, and 10 were distinct from all other suppliers, they could not be assigned to a specific species because of a lack of reference data. Supplier 4 was attributed to *S. procumbens*, and supplier 5 was once more categorized as *Sarcocornia fruticosa*, *Sarcocornia perennis*, or *Sarcocornia pruinosa*. Supplier 7 was verified as *Arthrocnemum macrostachyum*. Notably, the ambiguous assignment of supplier 3 remained.

#### 3.2.3 *matK* tree evaluation

The phylogenetic tree derived from the *matK* marker is available in an additional file (**Supplementary Table S7**; **Figure 3**). The sequences from suppliers 1, 2, 3, 8, and 11 are located within a complex clade that is most closely associated with reference sequences of *S. europaea*, *S. obscura*, *S. ramosissima*, *S. disarticulata*, and *S. x marshallii*. Notably, two supplier 8 sequences were also found in a poorly supported clade alongside reference sequences of *S. dolichostachya*, *S. obscura*, *S. emerici*, *S. persica*, *S. patula*, and *S. veneta*. Furthermore, this main clade was not clearly differentiated from the reference sequences of *S. uniflora* and *S. brachiata*. All sequences from suppliers 6, 9, and 10, on the other hand, were situated in a clade that showed only weak differentiation (32% bootstrap probability) from all the aforementioned species. Collectively, all these previously mentioned sequences form a common, well-defined clade that is clearly distinct from the clade containing all the sequences of supplier 4 along with the reference sequences of *S. dolichostachya* and *S. emerici*. This entire group of species is also unequivocally separated (100% bootstrap probability) from all sequences of supplier 5, which align with a reference sequence of *S. fruticosa*, with an *S. perennis* reference sequence also located nearby. Finally, this combined group of *Sarcocornia* and *Salicornia* sequences was most distantly related to the sequences of supplier 7, which clustered exclusively with a reference sequence of *Arthrocnemum glaucum* in a highly supported group (100% bootstrap probability) that was also distinct from the adjacent reference sequence of *Arthrocnemum macrostachyum*. The analysis successfully delineated suppliers 1, 2, 3, 8, and 11 from other suppliers. However, unambiguous assignment to *S. europaea* was not possible in this analysis because of the presence of additional taxa within the same cluster. The same limitation applied to suppliers 6, 9, and 10, although without reference sequences present in their cluster. Supplier 4 was assigned to the *S. procumbens* taxon, supplier 5 was identified as *Sarcocornia fruticosa* (or possibly *S. perennis*), and supplier 7 was determined to be *Arthrocnemum macrostachyum* (likely synonymous with *Arthrocnemum glaucum*). Moreover, subspecies identification is unfeasible with *matK* because this level of detail is not provided in the reference sequences.

#### 3.2.4 *atpB-rbcL* tree evaluation

**Figure 4:**
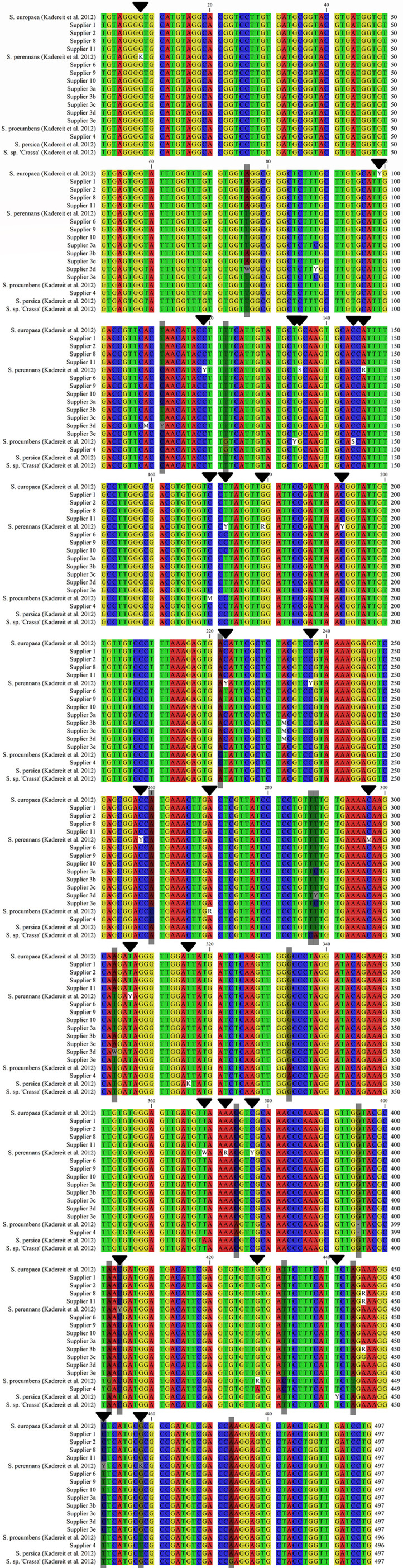
Consensus sequence alignment of *S. europaea*, *S. perennans*, *S. procumbens*, and *S. persica* (Kadereit et al., 2012) and *Salicornia* sp. [S. “Crassa” group (Kadereit et al., 2007)] with representative sequences of the investigated samples (despite the use of all five variable sequences from supplier 3). Variable sites are marked with black arrows, and diagnostic sites are shaded gray. A colon (:) represents a deletion/indel.

The phylogenetic tree derived from the *atpB-rbcL* marker is available in an additional file (**Supplementary Table S7**; **Figure 4**). All sequences from suppliers 1, 2, 3, 4, 6, 8, 10, and 11 are located within a largely unresolved cluster, displaying only weakly supported internal subclusters. This primary clade is most closely associated with reference sequences of *S. europaea*, *S. x tashkensis*, *S. persica*, *S. perspolitana*, *S. iranica*, *S. procumbens*, and *S. perennans*. Furthermore, it also includes reference sequences of *S. iranica* subsp. *rudshurensis*, *S. iranica* subsp. *iranica*, *S. iranica* subsp. *sinus-persica*, and *S. turanica* and is clearly differentiated from only a single reference sequence of *S. procumbens* subsp. *heterantha* (93% bootstrap probability). This entire large clade is, in turn, distinguishable from a phylogenetically more distant clade, which itself was divided into two subclades (56% bootstrap probability). One of these subclades contains all sequences from supplier 5 along with reference sequences from all included *Sarcocornia* species, such as *S. alpini* and *S. fruticosa*. This combined *Sarcocornia* and *Salicornia* group is clearly separated into a clade containing the sequences of supplier 7 and a reference sequence of *Arthrocaulon macrostachyum*. In summary, only supplier 5 could be unambiguously identified as *Sarcocornia fruticosa* (or a subspecies of *Sarcocornia perennis*), and supplier 7 could be identified as *Arthrocnemum macrostachyum*. Furthermore, subspecies classification is also not possible for *atpB-rbcL*, as these distinctions are absent in the reference data.

### 3.3 SNP analysis

Variable and diagnostic nucleotide positions of the ETS marker, which characterize the different *Salicornia* taxa, have been reported (Kadereit et al., 2012) and evaluated for congruence and clarity. Diagnostic positions 76, 111, and 303, characteristic of *S. europaea*, were found in the sequences of suppliers 1, 2, 8, and 11 and partially in those of supplier 3 (**Figure 4**). *S. procumbens* is characterized by five variable (134, 144, 170, 270, and 428) and nine diagnostic (122, 221, 260, 333, 374, 395, 402, 432, and 444) nucleotides (Kadereit et al., 2012). The sequences from supplier 4 uniquely exhibited this combination, whereas they were not found for any other supplier (**Figure 4**). For *S. perennans*, while 18 variable positions have been reported, no synapomorphic mutation has been identified (Kadereit et al., 2012). A synapomorphy is a shared, derived character that is common between an ancestor and its descendants. Consistent with their assignment to *S. perennans*, suppliers 6, 9, and 10 presented no contradictions at these variable positions or in the remaining sequence data (**Figure 4**). Furthermore, these suppliers were clearly assigned to *S. perennans* by distinguishing diagnostic positions from other species, including *S. europaea* (positions 76, 111, 303) and *S. procumbens* (positions 122, 221, 260, 333, 374, 395, 402, 432, 444). Differentiation from *S. persica* relies on a verified diagnostic nucleotide at position 404 and differences in variable, nonmatching nucleotides at positions 369 and 458 (**Figure 4**; (Kadereit et al., 2012). Further differentiation from *S.* sp. (“Crassa” group) utilized three diagnostic nucleotides (positions 286, 287, 473) (Kadereit et al., 2012), which were exclusive to the “Crassa” group, confirming that there was no homology with sequences from suppliers 6, 9, and 10 (**Figure 4**).

The samples from supplier 3 presented mixed and ambiguous homology to *S. europaea* and *S. perennans* sequences. Samples 3b and 3c were homologous to *S. europaea* on the basis of their diagnostic nucleotides and could not be differentiated from *S. europaea* by other diagnostic nucleotides (**Figure 4**). Sample 3d displayed three variable nucleotides (**Figure 4**) intermediate between those of *S. europaea* and other *Salicornia* taxa at diagnostic positions for *S. procumbens*, *S. persica*, and *Salicornia* sp. (“Crassa” group). Samples 3a and 3e differed from *S. europaea*, *S. procumbens*, *S. persica*, and *Salicornia* sp. (“Crassa” group) at the aforementioned diagnostic positions. Consequently, samples 3a, 3d, and 3e were more likely homologous to *S. perennans* sequences (**Figure 4**).

Taken together, the analysis revealed distinct taxonomic assignments for most suppliers. Suppliers 1, 2, 8, and 11 were largely assigned to the *S. europaea* taxon, whereas supplier 4 was unambiguously identified as belonging to the *S. procumbens* taxon. Suppliers 6, 9, and 10 were attributed to the *S. perennans* taxon. Notably, supplier 3 could not be definitively assigned to either the *S. europaea* or *S. perennans* taxon. These clear assignments to the five main taxa were supported by diagnostic positions from the SNP analysis.

### 3.4 RNA topology analysis

**Figure 5:**
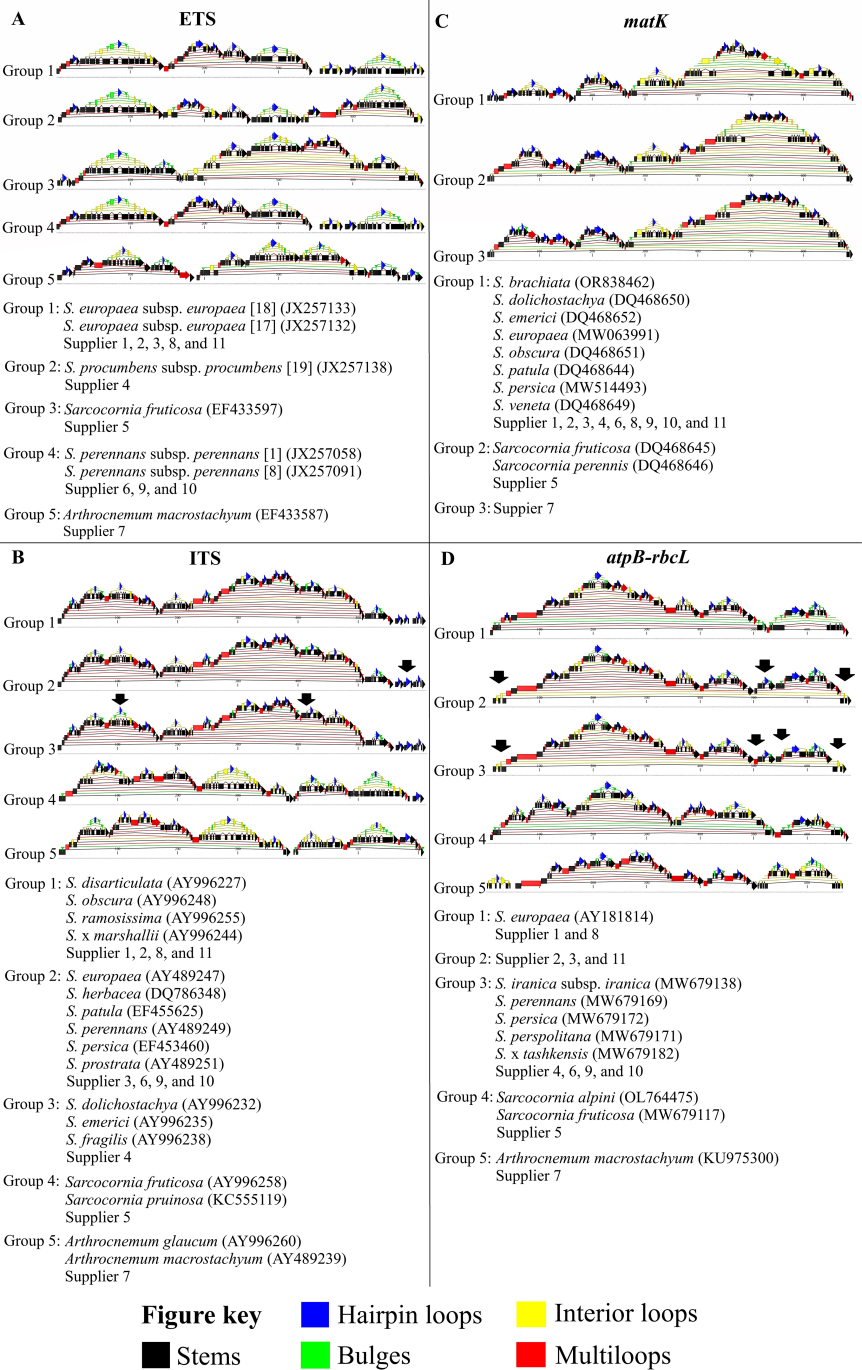
RNA topology of the **(A)** partial genomic region of the external transcribed spacer and small subunit ribosomal RNA, **(B)** partial genomic region of the internal transcribed spacer 1, 5.8S ribosomal RNA, and internal transcribed spacer 2, **(C)** partial chloroplast region of the *matK* gene, and **(D)** partial chloroplast region of the *atpB-rbcL* intergenic spacer, visualized for the core sequences of all suppliers and eligible candidate reference sequences. The samples were ordered by supplier number and grouped on the basis of topological homology to enhance visualization and identify relationships between the sequences derived from different suppliers.

An analysis of the predictions for RNA secondary structures of the ETS region revealed that the structures from suppliers 1, 2, 3, 8, and 11 presented identical features and were homologous to reference sequences of *S. europaea* subsp. *europaea* (ribotypes 17 and 18), summarized as group 1 (**Figure 5A**). The ETS RNA secondary structures of supplier 4 were homologous to each other and to reference sequences of *S. procumbens* subsp. *procumbens* (ribotype 1), forming group 2 (**Figure 5A**). Supplier 5 displayed ETS RNA secondary structures corresponding to those of reference sequences from *Sarcocornia fruticosa*, represented as group 3 (**Figure 5A**). In contrast, the ETS RNA secondary structures of suppliers 6, 9, and 10 showed sufficient topological differences to warrant separate categorization and matched references of *S. perennans* subsp. *perennans* (ribotypes 1 and 8), summarized as group 4 (**Figure 5A**). Furthermore, the ETS RNA secondary structure predictions for supplier 7 formed a distinct group (group 5), matching a reference of *Arthrocnemum macrostachyum* (**Figure 5A**).

The ITS RNA secondary structure predictions resulted in the same number of groups as those for the ETS region (**Figure 5B**). Group 1 comprised suppliers 1, 2, 8, and 11, along with references from *S. disarticulata*, *S. obscura*, *S. ramosissima*, and *S. x marshallii*, but no sequences from supplier 3. In contrast, group 2 contained the RNA secondary structures of supplier 3, as well as those of suppliers 6, 9, and 10, together with references from *S. europaea*, *S. herbacea*, *S. patula*, *S. perennans*, *S. persica*, and *S. prostata*. Group 3 included the predictions for supplier 4 and references from *S. dolichostachya*, *S. emerici*, and *S. fragilis*. The ITS RNA secondary structure of supplier 5 (group 4) again matched references from *Sarcocornia fruticosa* and *Sarcocornia pruinosa*. Finally, the RNA secondary structure prediction for supplier 7 (group 5) was congruent with references from *Arthrocnemum macrostachyum* and *Arthrocnemum glaucum*.

Furthermore, the RNA secondary structure predictions for *matK* did not allow for a subdivision of the *Salicornia* species (**Figure 5C**). All sequences from suppliers 1, 2, 3, 4, 6, 8, 9, 10, and 11 resulted in homogeneous secondary structures, which matched references from *S. brachiata*, *S. dolichostachya*, *S. emerici*, *S. europaea*, *S. obscura*, *S. patula*, *S. persica*, and *S. veneta* (group 1). For the reference sequences of *Sarcocornia fruticosa* and *Sarcocornia perennis*, secondary structures that were homologous to those of supplier 5, summarized as group 2, were predicted (**Figure 5**). The analysis of the *matK* RNA secondary structures for supplier 7 (group 3) revealed clear differences from all other sequence predictions (**Figure 5C**). The highest visual congruence was observed with a reference from *Arthrocnemum macrostachyum*, whereas the structure prediction for *Arthrocnemum glaucum* was distinctly different.

For the RNA structure predictions of the *atpB-rbcL* intergenic spacer (**Figure 5D**), the sequences from suppliers 1 and 8 were identified as identical to the prediction for a reference of *S. europaea* (group 1). The RNA structures of suppliers 2, 3, and 11 formed a distinct group (group 2), which clearly differed from those of group 1 but contained no homologous references. References from *S. iranica* subsp. *iranica*, *S. persica*, *S. perspolitana*, and *S. x tashkensis*, along with the secondary structure predictions for suppliers 4, 6, 9, and 10, formed a common homologous group (group 3). The secondary structure prediction for supplier 5 was, in turn, homologous to references from *Sarcocornia alpini* and *Sarcocornia fruticosa* (group 4). For supplier 7, an RNA secondary structure emerged (group 5) that was homologous to a reference sequence of *Arthrocnemum macrostachyum* and differed from that of an *Arthrocnemum subterminale* reference.

The ETS marker provided clear references within the five identified groups, unequivocally classifying the respective suppliers. While the ITS marker also delineated five groups with the same supplier assignments, it could not make a clear species assignment for the second group. The *matK* marker distinguished only three groups and could confirm the assignment of supplier 5 to *Sarcocornia fruticosa* or *Sarcocornia perennis* within the second group. The *atpB-rbcL* marker differentiated suppliers 1 and 8 from suppliers 2, 3, and 11 on the basis of marginal differences, but it failed to unequivocally assign the latter group to *S. europaea*. Furthermore, no clear species assignment was possible for group 3. In contrast, group 4 was clearly assigned to *Sarcocornia fruticosa* or a subspecies of *Sarcocornia perennis*, and group 5 was assigned to *Arthrocnemum macrostachyum*.

### 3.5 SSR analysis

**Figure 6:**
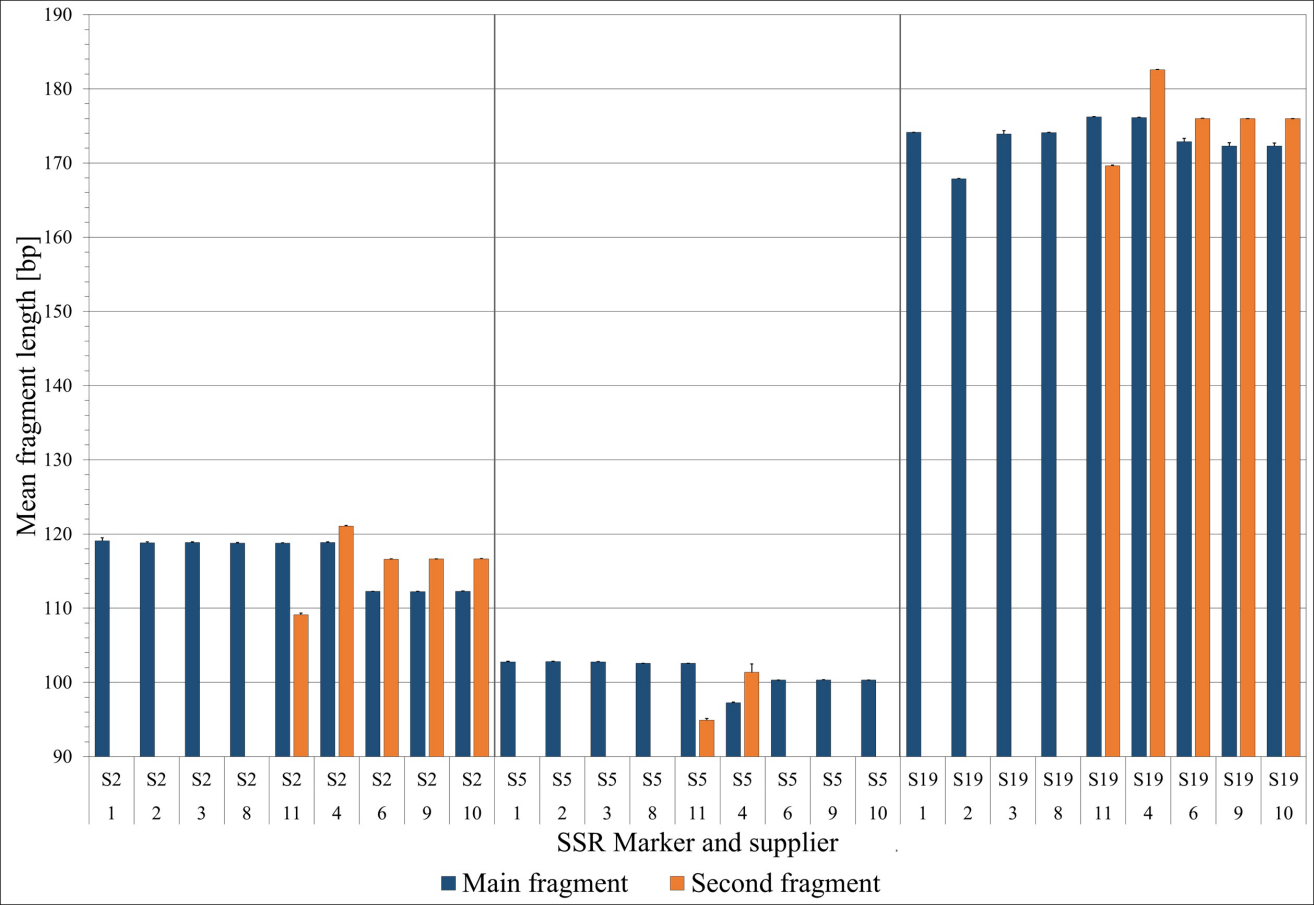
Mean specific fragment lengths (+ SD) across the S2, S5, and S19 microsatellite (SSR) markers (first row of the x-axis labeling) for each supplier (second row of x-axis labeling). If a second fragment was observed in the majority of samples for a particular marker, its mean length was also included.

To evaluate their potential for species discrimination, the S2, S5, and S19 microsatellite (SSR) markers were analyzed for patterns of homozygosity/heterozygosity and the presence of specific fragment lengths, which demonstrated varying degrees of resolution in distinguishing among *Salicornia* species (**Figure 6**). An additional table presents the results of the three markers for five samples from each supplier, with the exception of suppliers 5 and 7, which did not yield clear PCR amplicons (**Supplementary Table S5**). As shown in **Figure 6**, the S19 marker consistently distinguished the samples from suppliers 1 and 8 from supplier 2 and from supplier 11. Suppliers 1 and 8 in particular had a characteristic fragment length of 174 bp, whereas supplier 2 presented a clear fragment length of 167 bp, and supplier 11 presented 176 bp (with the exception of one sample with only 167 bp), whereas some samples from supplier 11 additionally presented smaller fragments 583 (**Supplementary Table S5**). Supplier 11 was further delimited by the fact that a second fragment of unique length was found in the majority of the samples in both the S2 and S5 markers, although this was also true in parts of the supplier 8 samples (**Supplementary Table S5**). Supplier 3 showed that the S2, S5 and S19 markers were mostly the same fragment size as suppliers 1 and 2 (**Supplementary Table S5**). **Figure 6** clearly shows that supplier 4 was differentiated both by fragment size and by the presence of two alleles (S2: 119 and 121 bp; S5: 97 and 100/102 bp; S19: 176 and 183 bp). Similarly, suppliers 6, 9, and 10 presented distinct allele numbers and fragment sizes across all three markers (S2: 112 and 117 bp; S5: 100 and partly 103 bp; S19: 172/173 and 176 bp) (**Figure 6**). Overall, only the S19 marker revealed further distinct differences, specifically separating suppliers 1 and 8 from supplier 2 and supplier 2 from supplier 11, beyond what was previously identified.

### 3.6 Nuclear DNA content determination and ploidy estimation

The flow cytometry measurement histograms for all samples, along with the utilized references, exhibited clear peak patterns and were averaged over three replicates. An additional table shows representative histograms for each supplier and for the *Trifolium pratense* reference (**Supplementary Table S6**). The nuclear DNA content of *Trifolium pratense* ‘Bingenheimer’ was determined to be 0.92 pg/2C and was used as a reference for all *Salicornia* species. On the basis of the size of the haploid genome and in comparison with literature data (Zonneveld, 2019), assumptions were made about the ploidy level of *Salicornia* species, which consistently have 9 chromosomes (basic chromosome number) (Zonneveld, 2019). The samples of all suppliers displayed endopolyploidy, a phenomenon already known for other Chenopodioideae species (**Supplementary Table S6**) (Skaptsov et al., 2017).

Suppliers 1 and 8 were classified as diploids on the basis of their DNA content and presented very similar genome sizes of 565.4 Mbp and 579.1 Mbp, respectively. Similarly, suppliers 2 and 11 were classified as diploid with slightly larger genome sizes of 594.1 Mbp and 568.7 Mbp, respectively. The diploid genome size of supplier 3, 596.7 Mbp, also fell within the range observed for the previously mentioned suppliers (**Table 2**).

**Table 2:** Summary of the cytometric characteristics of nine *Salicornia* samples, one *Sarcocornia* sample and one *Arthrocnemum* sample cultivated for this study.

S	Species	I	M	x	2n	Ploidy	References (2n and ploidy)	R	Mean DNA content [pg]	Genome size [Mbp/1C]
	*Trifolium pratense*	3	PI	7	14	2	(Vižintin et al., 2006)	*L*	0.92 ± 0.02	450.3
1	*Salicornia europaea* subsp. *europaea* cv. 1	3	DA	9	18	2	(Zonneveld, 2019)	*T*	1.16 ± 0.01	565.4
2	*Salicornia europaea* cv. 1	3	DA	9	18	2	(Zonneveld, 2019)	*T*	1.21 ± 0.03	594.1
3	*Salicornia europaea* x *perennans*	3	DA	9	18	2	(Zonneveld, 2019)	*T*	1.22 ± 0.02	596.7
4	*Salicornia procumbens* subsp. *procumbens*	3	DA	9	36	4	(Zonneveld, 2019)	*T*	2.39 ± 0.03	585.5
5	*Sarcocornia fruticosa*	3	DA	9	54 (36-72)	6 (4-8)	(Shepherd and Yan, 2003; Koce et al., 2008; La Fuente et al., 2016)	*G*	6.35 ± 0.4	1035.6 [6C] 776.3 [8C]
6	*Salicornia perennans* subsp. *perennans* cv. 1	3	DA	9	18	2	(Zonneveld, 2019)	*T*	2.14 ± 0.02	1047.5
7	*Arthrocnemum macrostachyum*	3	DA	9	36	4	(Shepherd and Yan, 2003; Lomonosova et al., 2020)	*G*	5.97 ± 0.1	1460.1
8	*Salicornia europaea* subsp. *europaea* cv. 2	3	DA	9	18	2	(Zonneveld, 2019)	*T*	1.18 ± 0.02	579.1
9	*Salicornia perennans* subsp. *perennans* cv. 2	3	DA	9	18	2	(Zonneveld, 2019)	*T*	1.98 ± 0.09	966.5
10	*Salicornia perennans* subsp. *perennans* cv. 3	3	DA	9	18	2	(Zonneveld, 2019)	*T*	2.09 ± 0.02	1021.3
11	*Salicornia europaea* cv. 2	3	DA	9	18	2	(Zonneveld, 2019)	*T*	1.16 ± 0.03	568.7

S: number of suppliers; Species: expected species and the corresponding cultivar; I: number of individuals analyzed; M: Method of PI = Propidiumiodide CyStain^®^ PI Absolute P, or DA = DAPI CyStain^®^ UV Precise P flow cytometric analysis; x: basic chromosome number according to a given reference; 2n: chromosome number known from the literature; Ploidy: ploidy level known from the literature; Reference (2n and ploidy): references for chromosome number and ploidy level; R: reference standard used in flow cytometry analyses, i.e., *G* − *Glycine max* cv. ‘Cina 5202’ [2.23 pg/2C], *L* − *Lycopersicon esculentum* convar. infiniens Lehm. var. flammatum Lehm., cv. ‘Stupicke Rane’ [1.96 pg/2C], *T* − *Trifolium pratense* cv. ‘Bingenheimer’ [0.92 pg/2C]; mean DNA content [pg]: mean measured 2C DNA content in picogram ± standard deviation; Mbp: calculated haploid genome size in megabase pairs (Mbp).

Owing to the known larger genome size (Zonneveld, 2019) of *S. perennans*, suppliers 6, 9, and 10 were classified as diploid representatives of this species group, with calculated genome sizes of 1047.5 Mbp (supplier 6), 1021.3 Mbp (supplier 9), and 926.9 Mbp (supplier 10), respectively (**Table 2**).

For supplier 4, the highest DNA content among the presumed *Salicornia* samples was measured. This value is typical for tetraploid species, leading to a calculated (haploid) genome size of 585.5 Mbp (**Table 2**).

For supplier 5, the ploidy level could not be definitively resolved because the reported haploid genome sizes in the literature are not particularly precise and because of the documented occurrence of variable ploidy levels within the genus. Compared with the reference data, a hexaploid level was presumed, yielding a calculated haploid genome size of 1035.6 Mbp (**Table 2**). However, on the basis of reference data suggesting a monoploid genome size of approximately 0.985 pg DNA content (Koce et al., 2008), an octoploid level could not be excluded. This alternative ploidy would imply a monoploid genome with approximately 0.106 pg of DNA content, corresponding to a genome size of 776.7 Mbp.

For supplier 7, a tetraploid ploidy level was expected (Shepherd and Yan, 2003), resulting in a potential haploid genome size of 1460.1 Mbp (**Table 2**). Importantly, an as-yet unrecorded hexaploid genome for the species could also be a factor, potentially leading to a genome size of 973 Mbp. suppliers 1, 2, 3, 8, and 11 presented the smallest genome contents typical of *S. europaea*. In contrast, supplier 4 was differentiated by a significantly larger genome content, characteristic of tetraploid species and known for *S. procumbens*. This also distinguished supplier 4 from supplier 6, 9, and 10, which presented an intermediate genome content typical of diploid species within the *S. perennans* taxon. Supplier 5 presented a considerably greater genome content, aligning more with the genus *Sarcocornia*. Finally, supplier 7 could not be assigned to any species on the basis solely of this analysis owing to a lack of reference data.

## 4 Discussion

The taxonomy within the genera *Salicornia*, *Sarcocornia*, and *Arthrocnemum* is characterized by significant complexity, as evidenced by numerous cases of heterotypic and homotypic synonymy (Govaerts et al., 2021). Furthermore, instances of hybridization, such as that of *S. x marshallii* (Stace et al., 2015; Stace, 2019), contribute to the intricate relationships among these groups. Understanding and accounting for this multifaceted nomenclature is crucial for the accurate interpretation of phylogenetic analyses involving these plant taxa.

Morphological analysis of the *Salicornia* samples revealed limited utility for unambiguous species identification. While vegetative characteristics and growth habits allowed tentative assignments on the basis of existing keys (Kadereit et al., 2012), the similar habits and subtle floral differences observed across several suppliers (1, 2, 3, 6, 8, 9, 10) underscore the challenges of relying solely on morphology. Furthermore, the dependence of identification keys on geographic distribution, an inapplicable criterion for sourced seed material, highlights inherent uncertainty. The inability to induce flowering in suppliers 5 and 7 further restricted morphological assessment to vegetative traits alone. Distinguishing taxa and differentiating the species on the basis of morphological identification keys partly relies on their geographic distribution. However, this criterion is limited to wild-collected samples and is not applicable to seed samples obtained from various suppliers. These findings collectively suggest that morphological data, while providing some discriminatory power (e.g., growth habit differences and inflorescence length in supplier 4), are often insufficient for definitive species delimitation within this complex, necessitating the integration of molecular data for more robust taxonomic resolution (Almuteri et al., 2025; Zaman et al., 2025).

Phylogenetic tree evaluation provided valuable insights into species relatedness. The ETS tree showed high consistency due to the already verified *Salicornia* reference sequences used for construction, although the subspecies were mostly indistinguishable. Additionally, the paraphyletic nature of the *Salicornia perennans* was confirmed, which comprises at least two distinct clades (Kadereit et al., 2012). One of these clades (including ribotypes 1, 5, 8, 9, 10, 11, 12, 13, 14, 15, and 25), with the exception of ribotype 5, has not been previously detected in mainland Europe; it primarily occurs in Southeast Europe to Asia. This finding suggests a taxonomic division of *S. perennans* clades that relies more on geographical distribution than on genetic and morphological differences. Therefore, the classification of samples from suppliers 6, 9, and 10 (and partially supplier 3) within the *S. perennans* species group should be interpreted cautiously. Further taxonomic refinement of this group, using improved methods, is warranted. RNA structure prediction refined sample identification: suppliers 1, 2, 3, 8, and 11 consistently matched *S. europaea* subsp. *europaea*. Supplier 4 was unambiguously identified as *S. procumbens* subsp. *procumbens* [ribotype 19], which is consistent with its Western European distribution (Kadereit et al., 2012). Supplier 5 matched only *Sarcocornia fruticosa*, refuting the prior *Sarcocornia perennis* assignment. Suppliers 6, 9, and 10 corresponded to *S. perennans* subsp. *perennans* [ribotypes 1 and 8], suggesting the potential introduction of East European/Asian lineages (Kadereit et al., 2012) in European markets. Supplier 7 exclusively matched *A. macrostachyum*, corroborating the ETS phylogeny. Although SNP analysis offered a more detailed confirmation of existing findings, its application was confined to known diagnostic positions for the taxa, specifically excluding suppliers 5 and 7. The consistent presence of diagnostic nucleotides (Kadereit et al., 2012) in suppliers 1, 2, 8, and 11 strongly supports their identification as belonging to the *S. europaea* taxon. Similarly, the exclusive match of sequences from supplier 4 to the diagnostic nucleotides of the *S. procumbens* taxon provides robust corroboration for its taxonomic assignment. Furthermore, through exclusion, suppliers 6, 9, and 10 were unambiguously assigned to the *S. perennans* taxon.

The SNP detection of nucleotide homologies in the supplier 3 sequences of both *S. europaea* and *S. perennans* across the analyzed samples suggests a novel hybridization event between these species. This genetic complexity indicates that the seedstock consists at least partly of hybrids and that genetic heterogeneity resulting from incomplete lineage sorting warrants further investigation.

The ITS tree also showed high distinctness but included sequences of other taxa, particularly within the *S. europaea* and *S. perennans* clusters, suggesting potentially misannotated database sequences that limit ITS marker reliability. Therefore, RNA structure analysis of ITS sequences is not suitable for species identification because of reference data limitations. Suppliers 1, 2, 8, and 11 exclusively matched *S. europaea*. Supplier 3 showed structural homology with suppliers 6, 9, and 10 and could not be differentiated from *S. europaea*, *S. perennans*, or *S. persica*. Supplier 4 matched *S. procumbens*. Supplier 5 coincided with *Sarcocornia fruticosa* and *Sarcocornia pruinosa*, leaving a definitive distinction unresolved. Supplier 7 corresponded to *A. macrostachyum* and *A. glaucum*. The joint use of ETS-ITS could not be verified because of insufficient matching reference sequences, which does not disprove its potential. The results of the *matK*-derived phylogeny were comparable to those of the ITS, with both lacking usable *S. perennans* references. Delimitation of *S. perennans* from *S. europaea* showed high uncertainty due to low bootstrap support. The RNA secondary structure prediction of *matK* sequences did not increase resolution. The *atpB-rbcL* marker was not useful for delimiting *S. persica*, *S. procumbens*, *S. perennans*, or *S. europaea*. *Sarcocornia* species delimitation had relatively low confidence. However, the *atpB-rbcL* intergenic spacer RNA structure allowed the discrimination of suppliers 1 and 8 from suppliers 2, 3, and 11, suggesting the potential for subspecies differentiation within *S. europaea*. Conversely, it did not differentiate suppliers 4, 6, 9, and 10.

In accordance with the literature, molecular markers can often be recommended for plant species determination (Chase and Fay, 2009), offering particular advantages over morphological methods in groups with high plasticity, convergence, or a lack of diagnostic features (Hollingsworth et al., 2011), as well as in situations involving hybridization and polyploidy that cause morphological variation and overlap (Soltis and Soltis, 2009). As observed in wild potatoes, the use of ITS and plastid markers alone is insufficient for species identification, suggesting that the integration of morphological and molecular techniques, alongside the selection of appropriate markers, is advisable for certain species (Spooner, 2009). Conversely, these markers have been successfully employed for species identification in other genera, such as *Pulsatilla* (Ranunculaceae) (Li et al., 2019), medicinal species of Fabaceae and Poaceae (Tahir et al., 2018), and terrestrial invasive plant species in Southwest Michigan (Nath et al., 2024). For *Salicornia* spp., the ETS region can be considered particularly informative for species identification, a finding that is consistent with prior evidence in the *Avena* genus (Rodrigues et al., 2017) and the Apiaceae tribe Tordylieae (Logacheva et al., 2010).

Microsatellite fragment length analyses (S2, S5, and S19) aimed to differentiate species within the *Salicornia* complex. While microsatellite markers on their own are generally not ideal barcodes for *Salicornia* (Vanderpoorten et al., 2009), fragment size differences show high consistency, making them useful for differentiating subspecies or ecotypes within an identified taxon. Consistent with the results obtained here, the discrimination of populations and partially of species and the prediction of specific attributes such as genotype flavonoid content have been reported for a range of plant species in previous research on SSR markers (Ali et al., 2019; Lebedev et al., 2019; Hamm et al., 2023; Liang et al., 2025). Locus S19 was the most informative, providing a robust diagnostic feature for resolving cryptic diversity. Locus S5 was least informative, and S2 was moderately useful. Building a S19 reference fragment length collection for molecular differentiation might be useful for further refining *Salicornia*’s taxonomy. For each supplier the species identification results of this investigation, the countries from which the seeds were obtained, and the methodologies deemed sufficient for species and subspecies delimitation are presented in the following summary (**Table 3**).

**Table 3:** Summary of species identification results for individual suppliers of European seed material obtained during this study.

S.	Country of sale	Species assignment	Morph-ology	Phylogenetic reconstruction				ETS SNP	RNA topology				SSR analysis			Nuclear DNA content	Summary
				ETS	ITS	*matK*	*atpB-rbcL*		ETS	ITS	*matK*	*atpB-rbcL*	S2	S5	S19		
1 & 8	Belgium	*S. europaea* subsp. *europaea*	Not suit.	++	*++*	*++*	Not suit.	++	+++	*++*	Not suit.	++	Same group except supplier 11	Same group except supplier 11	Distinct group	++	ETS barcoding (incl. SNP or RNA structure comparison) and fragment length analysis for further differentiation
2 & 11	Portugal	*S. europaea*	Not suit.	++	*++*	*++*	Not suit.	++	+++	*++*	Not suit.	Not suit.			Supplier 2 & 11 form distinct groups	++	
3	Belgium	*S. europaea* and *S. perennans* (likely hybrid)	Not suit.	Mixed species	Not suit.	*++*	Not suit.	Hybrid species	Mixed species	Not suit.	Not suit.	Not suit.			Mixed species	Not suit.	ETS barcoding (incl. RNA structure or partly SNP comparison)
4	France	*S. procumbens* subsp*. procumbens*	Not. suit.	+++	*+*	*+*	Not suit.	++	+++	*+*	Not suit.	Not suit.	Distinct group	Distinct group	Distinct group	++	
5	Portugal	*Sarcocornia fruticosa* (or potentially *S. pruinosa*)	Not appl.	+	Not suit.	++	+	Not appl.	++	+	+	+	Not appl.	Not appl.	Not appl.	Not suit.	
6, 9, & 10	Netherlands, Ireland, France	*S. perennans* subsp. *perennans*	Not suit.	+++	Not suit.	Not suit.	Not suit.	++	+++	Not suit.	Not suit.	Not suit.	Distinct group	Distinct group	Distinct group	++	
7	Portugal	*Arthrocnemum macrostachyum*	Not appl.	++	++	++	++	Not appl.	++	+	Not suit.	Species	Not appl.	Not appl.	Not appl.	Not suit.	

S.: number of suppliers; Country of sale: country from which the seeds were obtained; Species assignment: taxonomic identification of the European species; Morphology: Interpretation of morphological observations (3.1); Phylogenetic reconstruction: Interpretation of tree evaluation results for ETS, ITS, *matK*, and *atpB-rbcL* markers (3.2); ETS SNP: Interpretation of results for SNP analysis of ETS sequences (3.3); RNA topology: Interpretation of results for RNA topology analysis (3.4); SSR analysis: Interpretation of results for SSR analysis of S2, S5, and S19 markers (3.5); Nuclear DNA content: Interpretation of results for Nuclear DNA content determination and ploidy estimation (3.6); Summary: molecular methods required for accurate species and, if applicable, subspecies determination. Not suit. = the method was not suitable for species determination because of a lack of reference data. Not appl. = the method was not applicable because of a lack of data.

Genome size determination facilitates ploidy inference and taxonomic identification. This method relies on chromosome counts, which were acquired from literature. The analysis, which revealed a mean nuclear DNA content of 0.92 pg/2C for *Trifolium pratense* ‘Bingenheimer’, validated its utility as a reference for estimating the genome sizes of *Salicornia* species (**Table 2**). This result is consistent with previously reported data for other *Trifolium pratense* accessions (Vižintin et al., 2006). Despite identical chromosome numbers (2n=18) and confirmed diploidy, *S. perennans* and *S. europaea* exhibit substantial differences in diploid genome size, a phenomenon already reported in the literature (Zonneveld, 2019). This difference might be largely attributed to varying amounts of repetitive DNA, including transposable elements and tandem repeats, which are known drivers of genome size evolution in angiosperms (Bennetzen and Wang, 2014; Kelly et al., 2015). The larger genome of *S. perennans* therefore suggests greater accumulation of these elements, reflecting distinct evolutionary paths. These differences at the same ploidy level indicate the potential for species differentiation. Likewise, polyploid species, such as those from suppliers 4, 5, and 7, can be clearly distinguished by their genomic DNA content. However, relying solely on nuclear DNA content measurements is challenging, with limited reference data and variable ploidy levels.

This study’s findings are subject to certain limitations. The incomplete nature of existing reference databases for the markers used is a primary constraint, potentially affecting species identification and diversity assessment. Furthermore, potential hybridization events can complicate species delineation, and species synonymy within taxonomic classifications may introduce redundancies. Addressing these issues requires meticulous interpretation of phylogenetic results as well as ongoing efforts to expand databases and refine analytical methods. Therefore, the most robust approach integrates genome size and ploidy levels with molecular data. Our study highlights the efficacy of integrating RNA topology or SNP analysis of the ETS region, potentially obviating the need for additional morphological investigations. Microsatellite analysis revealed differences between suspected subspecies. However, its utility for standalone species identification is limited by a lack of reference data, necessitating further research. Nevertheless, it shows potential for distinguishing subspecies and populations, thus qualifying as a marker for targeted breeding. Future research should expand and refine genomic resources. Precisely resolving *Salicornia* taxonomy through advanced genotyping extends beyond commercial labeling, facilitating species-specific cultivation, bioproduct development, and ecological restoration. As previously recommended for horticulture (O Elansary et al., 2017), herbal drugs (Mishra et al., 2016), and the food industry (Galimberti et al., 2019), molecular methods should also be employed for the introduction of *Salicornia* for agricultural and nutritional purposes. ETS marker analysis has demonstrated the highest resolution and reliability, largely benefiting from a broad framework of verified sequences. It can also be implemented cost-effectively through simple sequence comparisons. Given the difficulty in differentiating subspecies via this marker, additional morphological characteristics or molecular identification tools, particularly fragment length analyses of promising microsatellite loci, need to be integrated for a more detailed analysis. **Table 3** summarizes the applicability and resolution of species assignment for each method used, including seed origin and the species identified by the most suitable, corroborating methods. It also provides recommendations for the applicable methodologies. Future research could explore the application of genome-wide association studies in promising *Salicornia* species. By analyzing genetic variation across a large collection of individuals and correlating it with specific qualitative traits (e.g., salt tolerance, branching patterns, or unique morphological features), this method could uncover novel genetic markers and provide deeper insights into the evolutionary biology of this genus, especially intraspecific differences (Visscher et al., 2017; He and Gai, 2023; Clauw et al., 2025).

## Data Availability

All sequence data and genome files generated for this study were deposited on NCBI under BioProject PRJNA1272992.
