# Supplementary material for: Comparative assessment of species identification methods for European Salicornia sources: a multifaceted approach employing morphology, nuclear DNA content, phylogenetic markers, RNA topology, and SSR fingerprinting
Source: Front Plant Sci. 2025 Sep 19;16:1666009. doi: 10.3389/fpls.2025.1666009 (PMC12491207; doi:10.3389/fpls.2025.1666009)
Supplement: Supplementary file 7 [file Table7.docx]

Supplementary Material


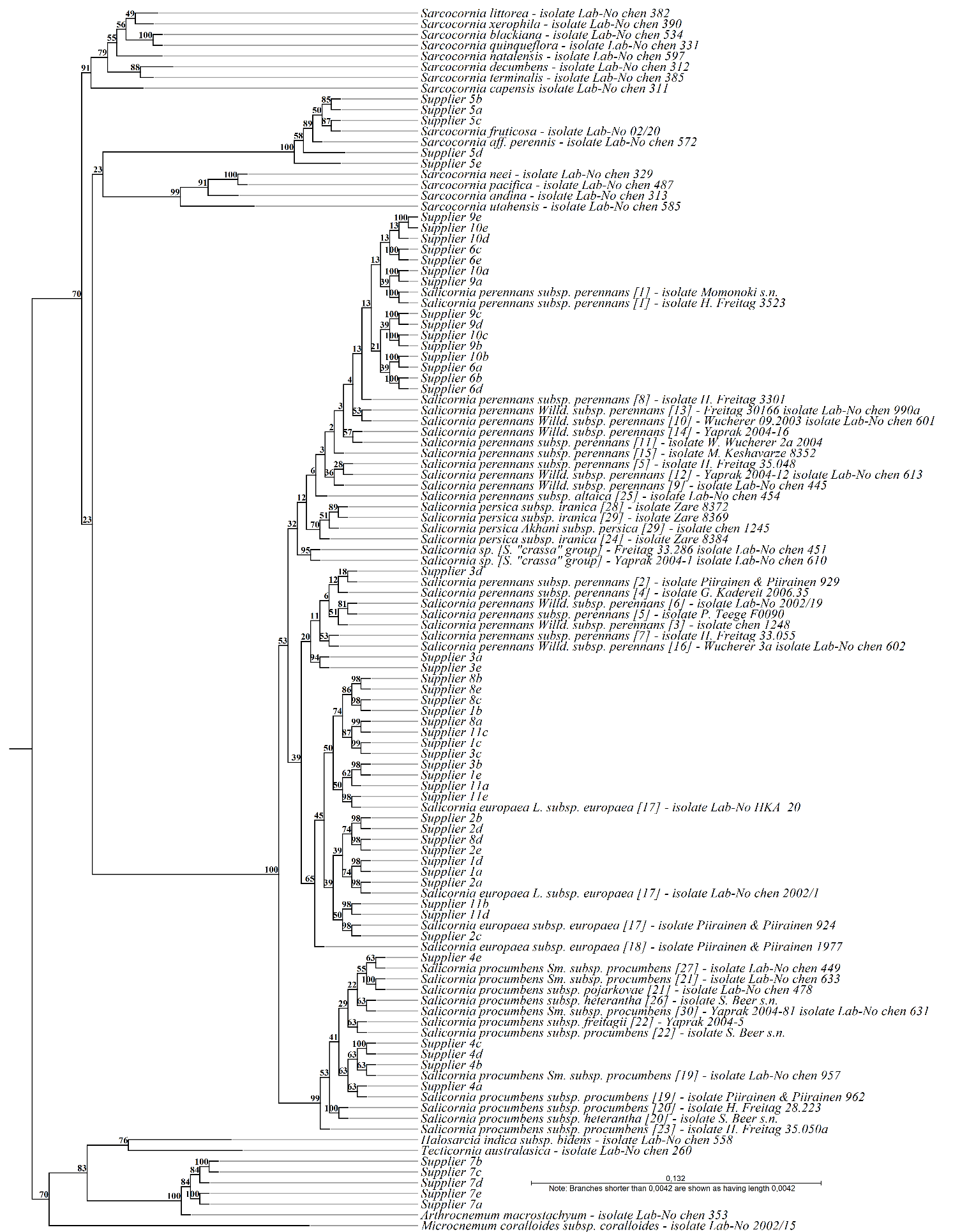


Supplementary Figure 1. The rooted maximum-likelihood (ML) phylogenetic ETS tree was inferred from the alignment of the genomic external transcribed spacer and small subunit ribosomal RNA partial sequences under the best-fit model GTR+G+T (selected by CLC Main Workbench hLRT, BIC, AIC, and AICc model testing). All known *Salicornia* ribotypes (Kadereit et al. 2012) were represented by at least one sequence, and the dataset was expanded with additional sequences from *Sarcocornia*, *Arthrocnemum*, and related species. The bootstrap values from 1000 resamplings are given at each node, and the branch lengths are stretched for easier representation, as indicated in the scale.


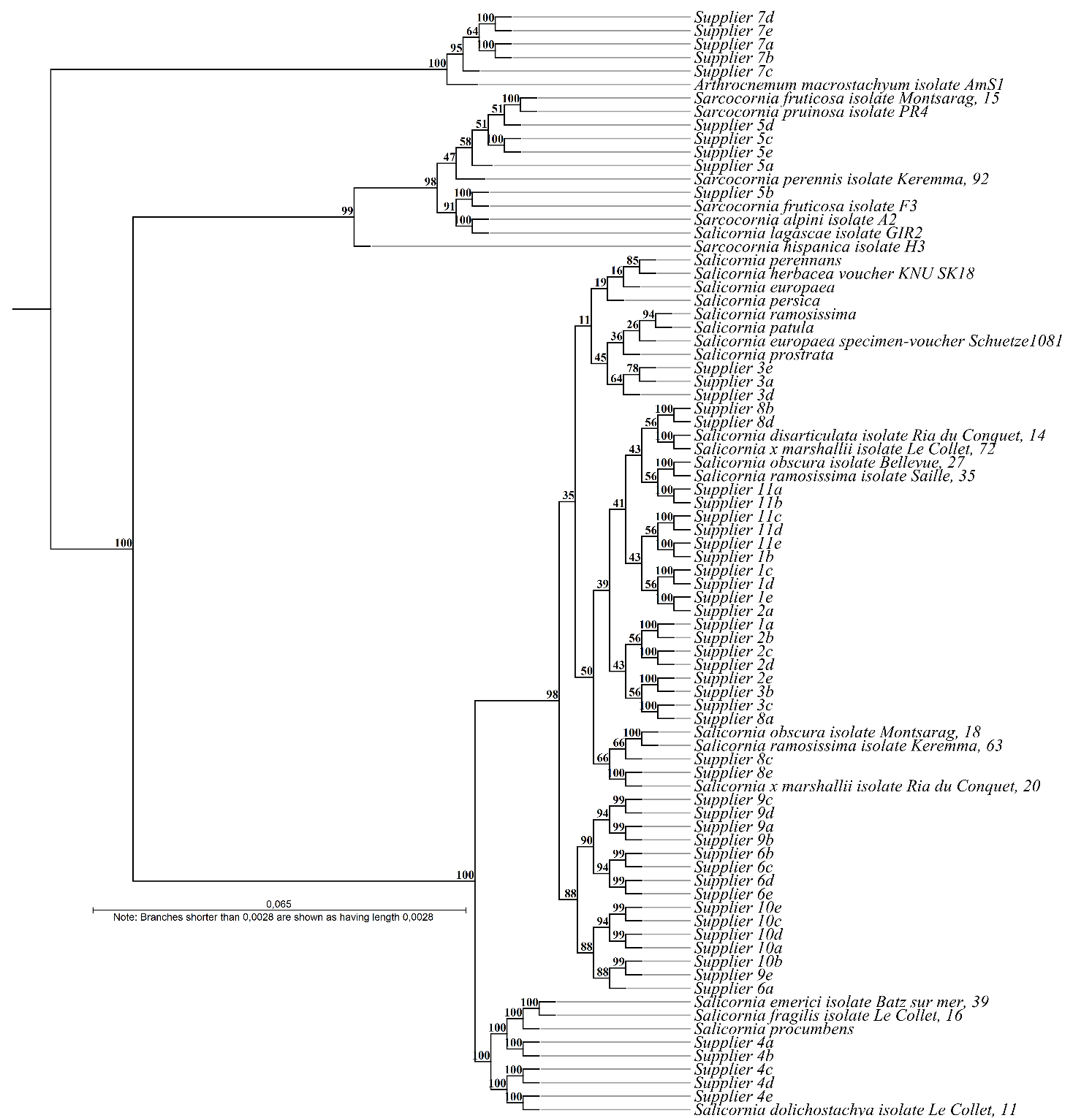


Supplementary Figure 2. The rooted maximum-likelihood (ML) phylogenetic ITS tree was inferred from the alignment of the genomic internal transcribed spacer 1, 5.8S ribosomal RNA, and internal transcribed spacer 2 partial sequences under the best-fit model GTR+G+T (selected by CLC Main Workbench hLRT, BIC, AIC, and AICc model testing). The bootstrap values from 1000 resamplings are given at each node, and the branch lengths are stretched for easier representation, as indicated in the scale.


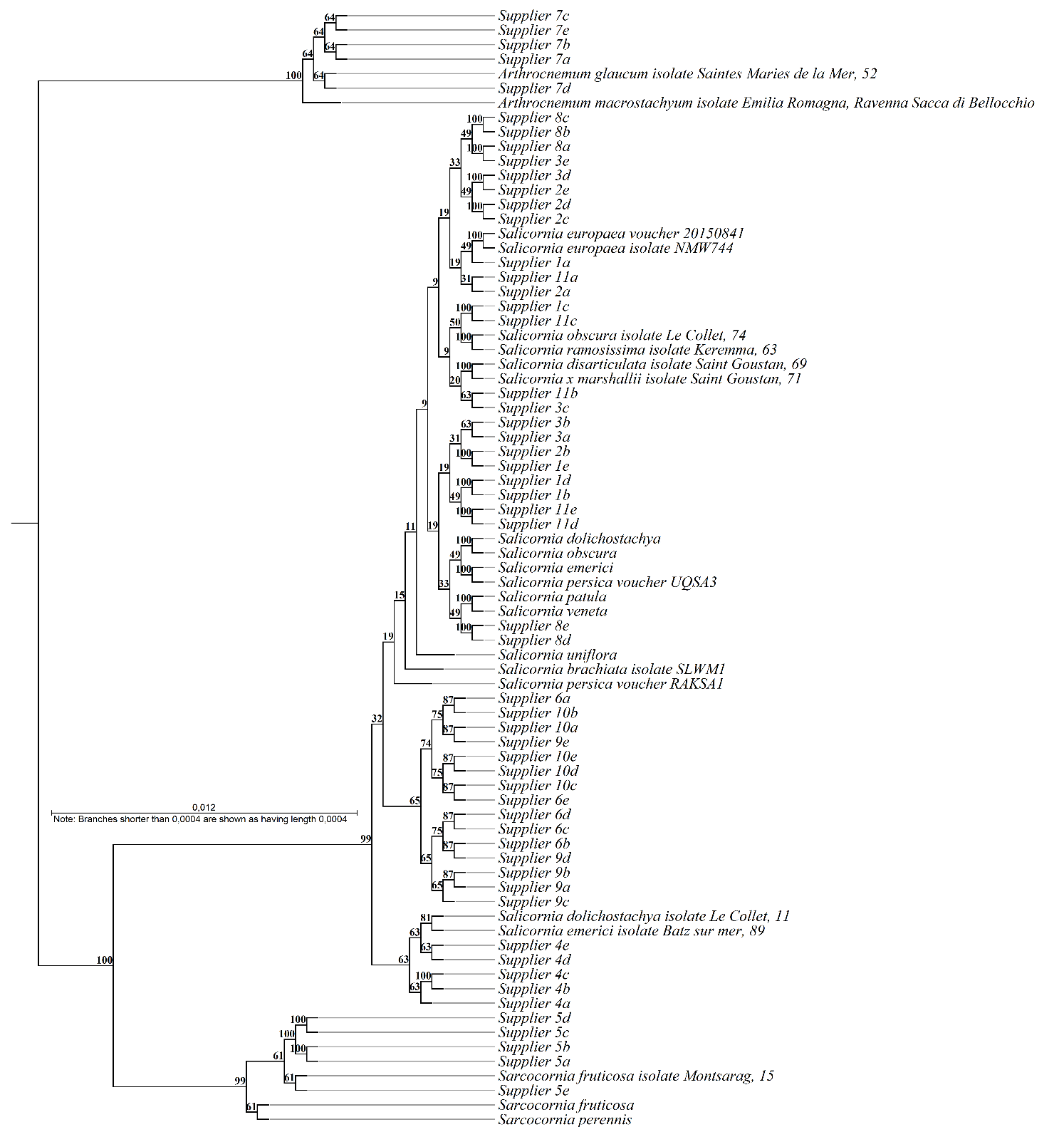


Supplementary Figure 3. The rooted maximum-likelihood (ML) phylogenetic *matK* tree was inferred from the alignment of the chloroplast matK partial gene sequences under the best-fit model F81+T (selected by CLC Main Workbench hLRT, BIC, AIC, and AICc model testing). The bootstrap values from 1000 resamplings are given at each node, and the branch lengths are stretched for easier representation, as indicated in the scale.


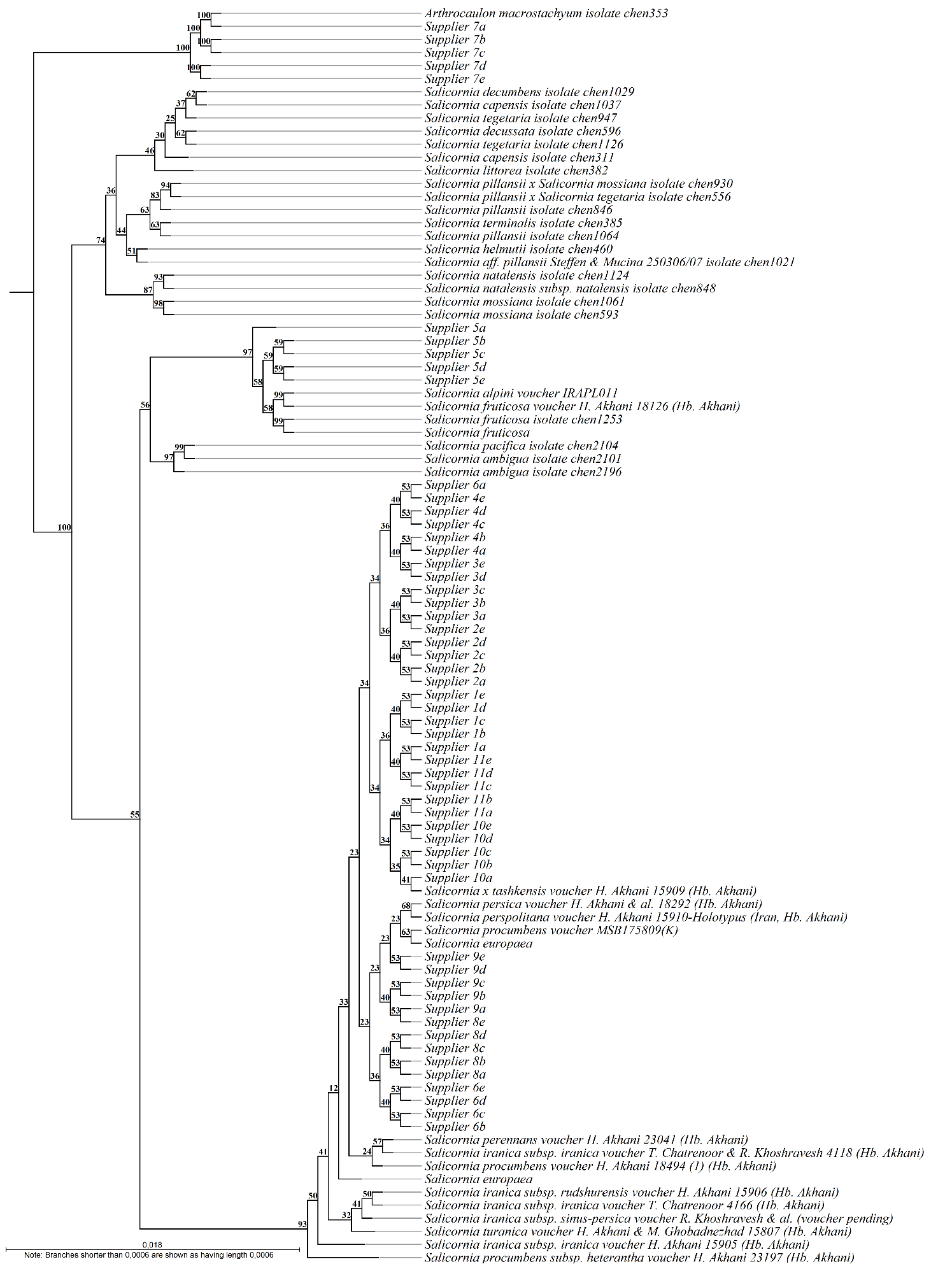


Supplementary Figure 4. The rooted maximum-likelihood (ML) phylogenetic *atpB-rbcL* tree was inferred from the alignment of the chloroplast atpB-rbcL intergenic spacer partial region sequences under the best-fit model F81+T (selected by CLC Main Workbench hLRT, BIC, AIC, and AICc model testing). The bootstrap values from 1000 resamplings are given at each node, and the branch lengths are stretched for easier representation, as indicated in the scale.
